# Demand creation for HIV testing services: A systematic review and meta-analysis

**DOI:** 10.1371/journal.pmed.1004169

**Published:** 2023-03-21

**Authors:** Anjuli D. Wagner, Irene N. Njuguna, Jillian Neary, Kendall A. Lawley, Diana K. N. Louden, Ruchi Tiwari, Wenwen Jiang, Ngozi Kalu, Rachael M. Burke, Dorothy Mangale, Chris Obermeyer, Jaclyn N. Escudero, Michelle A. Bulterys, Chloe Waters, Bastien Mollo, Hannah Han, Magdalena Barr-DiChiara, Rachel Baggaley, Muhammad S. Jamil, Purvi Shah, Vincent J. Wong, Alison L. Drake, Cheryl C. Johnson

**Affiliations:** 1 Department of Global Health, University of Washington, Seattle, Washington, United States of America; 2 Research & Programs, Kenyatta National Hospital, Nairobi, Kenya; 3 Department of Epidemiology, University of Washington, Seattle, Washington, United States of America; 4 University Libraries, University of Washington, Seattle, Washington, United States of America; 5 Department of Infectious Diseases Epidemiology, London School of Hygiene and Tropical Medicine, London, United Kingdom; 6 Clinical Research Department, London School of Hygiene and Tropical Medicine, London, United Kingdom; 7 Malawi Liverpool Wellcome Clinical Research Programme, Blantyre, Malawi; 8 The Global Fund to Fight AIDS, Tuberculosis and Malaria, Geneva, Switzerland; 9 Statistical Center for HIV/AIDS Research and Prevention, Fred Hutchinson Cancer Center, Seattle, Washington, United States of America; 10 Infectious and Tropical Diseases Department, Bichat-Claude Bernard Hospital, AP-HP, Paris, France; 11 Global HIV, Hepatitis and STI Programmes, World Health Organization, Geneva, Switzerland; 12 UNAIDS, Asia Pacific, Regional Support Team, Bangkok, Thailand; 13 USAID, Division of HIV Prevention, Care and Treatment, Office of HIV/AIDS, Washington DC, United States of America

## Abstract

**Background:**

HIV testing services (HTS) are the first steps in reaching the UNAIDS 95-95-95 goals to achieve and maintain low HIV incidence. Evaluating the effectiveness of different demand creation interventions to increase uptake of efficient and effective HTS is useful to prioritize limited programmatic resources. This review was undertaken to inform World Health Organization (WHO) 2019 HIV testing guidelines and assessed the research question, “*Which demand creation strategies are effective for enhancing uptake of HTS*?” focused on populations globally.

**Methods and findings:**

The following electronic databases were searched through September 28, 2021: PubMed, PsycInfo, Cochrane CENTRAL, CINAHL Complete, Web of Science Core Collection, EMBASE, and Global Health Database; we searched IAS and AIDS conferences. We systematically searched for randomized controlled trials (RCTs) that compared any demand creation intervention (incentives, mobilization, counseling, tailoring, and digital interventions) to either a control or other demand creation intervention and reported HTS uptake. We pooled trials to evaluate categories of demand creation interventions using random-effects models for meta-analysis and assessed study quality with Cochrane’s risk of bias 1 tool. This study was funded by the WHO and registered in Prospero with ID CRD42022296947.

We screened 10,583 records and 507 conference abstracts, reviewed 952 full texts, and included 124 RCTs for data extraction. The majority of studies were from the African (*N* = 53) and Americas (*N* = 54) regions. We found that mobilization (relative risk [RR]: 2.01, 95% confidence interval [CI]: [1.30, 3.09], *p* < 0.05; risk difference [RD]: 0.29, 95% CI [0.16, 0.43], *p* < 0.05, *N* = 4 RCTs), couple-oriented counseling (RR: 1.98, 95% CI [1.02, 3.86], *p* < 0.05; RD: 0.12, 95% CI [0.03, 0.21], *p* < 0.05, *N* = 4 RCTs), peer-led interventions (RR: 1.57, 95% CI [1.15, 2.15], *p* < 0.05; RD: 0.18, 95% CI [0.06, 0.31], *p* < 0.05, *N* = 10 RCTs), motivation-oriented counseling (RR: 1.53, 95% CI [1.07, 2.20], *p* < 0.05; RD: 0.17, 95% CI [0.00, 0.34], *p* < 0.05, *N* = 4 RCTs), short message service (SMS) (RR: 1.53, 95% CI [1.09, 2.16], *p* < 0.05; RD: 0.11, 95% CI [0.03, 0.19], *p* < 0.05, *N* = 5 RCTs), and conditional fixed value incentives (RR: 1.52, 95% CI [1.21, 1.91], *p* < 0.05; RD: 0.15, 95% CI [0.07, 0.22], *p* < 0.05, *N* = 11 RCTs) all significantly and importantly (≥50% relative increase) increased HTS uptake and had medium risk of bias.

Lottery-based incentives and audio-based interventions less importantly (25% to 49% increase) but not significantly increased HTS uptake (medium risk of bias). Personal invitation letters and personalized message content significantly but not importantly (<25% increase) increased HTS uptake (medium risk of bias). Reduced duration counseling had comparable performance to standard duration counseling (low risk of bias) and video-based interventions were comparable or better than in-person counseling (medium risk of bias). Heterogeneity of effect among pooled studies was high. This study was limited in that we restricted to randomized trials, which may be systematically less readily available for key populations; additionally, we compare only pooled estimates for interventions with multiple studies rather than single study estimates, and there was evidence of publication bias for several interventions.

**Conclusions:**

Mobilization, couple- and motivation-oriented counseling, peer-led interventions, conditional fixed value incentives, and SMS are high-impact demand creation interventions and should be prioritized for programmatic consideration. Reduced duration counseling and video-based interventions are an efficient and effective alternative to address staffing shortages. Investment in demand creation activities should prioritize those with undiagnosed HIV or ongoing HIV exposure. Selection of demand creation interventions must consider risks and benefits, context-specific factors, feasibility and sustainability, country ownership, and universal health coverage across disease areas.

## Abstract 2

Anjuli Dawn Wagner and colleagues systematically review and meta-analyse data from randomised clinical trials to explore the effectiveness of demand creation interventions for HIV testing services.

## Author summary

**Why was this study done?:**

- This review was undertaken to inform World Health Organization (WHO) 2019 and 2023 HIV testing guidelines.
- HIV testing services (HTS) is the first step in reaching global goals for HIV management and control.
- Evaluating the effectiveness of different demand creation interventions to increase uptake of HTS is useful to prioritize limited programmatic resources.

**What did the researchers do and find?:**

- We conducted a systematic review and meta-analysis to answer the question, “*Which demand creation strategies are effective for enhancing uptake of HTS*?” focused on populations globally.
- We found that several classes of demand creation interventions had important and significant impacts on HIV testing uptake. These included: mobilization, couple- and motivation-oriented counseling, peer-led interventions, conditional fixed value incentives, and short message service (SMS) interventions.
- We also found that reduced duration counseling and video-based interventions are an efficient and effective alternative to address staffing shortages.
- Box 1 provides a description of what each of these categories of demand creation interventions mean.

**What do these findings mean?:**

- These findings can allow policymakers to select effective demand creation strategies, prioritize resources efficiently, and de-prioritize less effective strategies for their own countries.
- The impact of an effective intervention may vary depending on where and how it is implemented; policy decisions should consider setting, feasibility and sustainability, country ownership, and universal health coverage.

## Introduction

The United Nations (UN) has set ambitious targets to have 95% of people living with HIV (PLWH) diagnosed, 95% of them on antiretroviral therapy (ART), and 95% of them virally suppressed by 2025 [1]. At the end of 2021, 85% of PLWH knew their status [2]. Despite substantial progress, gaps remain, with 7.8 million PLWH unaware of their status; additionally, there were still 1.5 million new HIV infections in the past year [2]. Those most affected by HIV remain unreached, particularly men and adolescents and young adults (aged 10 to 24) in southern Africa, key populations and their partners (including men who have sex with men [MSM], people who inject drugs [PWID], people in prisons and other closed settings, sex workers, and transgender people). Populations historically most affected by HIV have experienced societal marginalization, stigma, and low engagement with health care.

Demand creation includes activities intended to improve an individual’s knowledge and attitudes, motivation and intentions, and eventually decision and behavior to seek HIV testing services (HTS). Demand creation interventions include those intended to directly impact a barrier individuals may face in accessing HTS, for example, incentives, community mobilization campaigns, and counseling-oriented interventions. Interventions that have an indirect impact on a person’s demand for HTS or which might influence supply of, or access to HTS are not considered demand creation, for example, provider training, health service quality improvement, operational flow improvement, and novel HIV testing locations or modalities.

Closing the gaps and reaching the remaining PLWH who do not know their status will require generating demand for HTS among this population, as well as people at high ongoing risk and with limited access or uptake of health services. Resources for HIV funding dropped by US $1 billion in 2018, marking the first time that global HIV funding declined since 2000 [3]. In the face of plateauing and declining resources, prioritizing limited resources toward effective demand creation approaches is essential. Additionally, while supply side strategies for improving HIV testing uptake have improved coverage of HIV testing and testing frequency, the remaining populations may require re-prioritizing demand generation strategies.

We conducted a systematic review to assess which demand creation approaches for HTS were effective in order to provide clearer guidance to countries, programs, and key stakeholders. Findings of this review informed the World Health Organization’s (WHO) update to the consolidated guidelines on HTS [4] and are informing the update in 2023. Findings of the review can also be used to inform program planning.

## Methods

### Guiding frameworks

We followed the PRISMA guidance for the appropriate conduct and reporting of systematic reviews and meta-analyses. The review protocol was developed with input from University of Washington researchers, the WHO Guideline Development Group (GDG) and the WHO HIV Department. The full review protocol is available in Prospero with ID CRD42022296947 [5]. The review departed from the protocol in the following ways: (1) we did not include non-experimental studies, as there was sufficient data from randomized controlled trials (RCTs); and (2) we did not present GRADE tables.

To categorize demand creation strategies for HTS, we used the User-Centric Behavioral Framework, which blends the social-ecological framework (individual, personal, cultural/societal, and structural levels of influence) with the stages of change framework (unaware, aware, pre-intention, intention, action) [6]. This framework has previously been used to categorize demand creation strategies for voluntary medical male circumcision (VMMC), which has many of the same demand-related barriers as HIV testing. We used the framework to consider interventions that generated demand by moving individuals along the spectrum from unawareness to action, distinguishing demand generation from supply-side interventions. We categorized interventions using an inductive approach; major categories and subcategories were developed in collaboration with WHO and the GDG. Intervention categories were not mutually exclusive and included: (1) incentivization (subcategories: a: conditional financial and b: lottery); (2) mobilization; (3) tailored or targeted (a: peer-led, b: personalized content and messages, c: personal invitation letters); (4) messaging and counseling (a: general HIV counseling, b: HIV counseling plus economic empowerment, c: couple-oriented counseling, d: message content framing, e: motivation-oriented counseling, f: reduced duration or intensity counseling); and (5) digitization (a: video- or audio-based, b: social media, c: website, d: short message service [SMS]). See Box 1 for a table of definitions and descriptions of each intervention category.

**Box 1. Definitions of demand creation categories.**

**Table:** 

**Demand creation category**	**Types of interventions included**	**Definitions**
Incentivize	Incentives to clients or partners	Interventions categorized as incentives included any intervention that included the provision of resources (financial or non-financial) based on HIV testing uptake. This could include transfers of resources to parents or partners and couples either conditionally, including payments conditioned on HIV testing, or performance-based incentives, or unconditionally. The resource could include financial incentives (range: USD$1 to USD$50) or non-financial incentives such as household goods/supplies.
Mobilize	Activities aimed at increasing HIV uptake within specific communities	Interventions categorized as mobilization included a range of activities and materials (theater, sport, games, educational material, sermons, printed material) aimed at mobilizing community members to take up HIV testing. Mobilization occurred in a range of settings, including church or faith-based centers, non-faith-based community settings, and at locations used to seek sexual partners.
Tailor and target	Personalized content, peer-led, and personal invitation	Tailoring and targeting interventions are interventions that aim to overcome a specific barrier to HIV testing by leveraging affinity or tailored content. They may include peer-led programs or education targeted at specific concerns or barriers.
Counsel	Message framing, motivation-oriented, general counseling, couple-oriented counseling, and reduced duration counseling	Counseling interventions sought to understand how improvements in client-provider HIV testing counseling can improve uptake. Counseling interventions can include changes to message framing, implementation of motivation-oriented counseling, couples counseling, or approaches to shorten the duration of counseling.
Digitize	Mobile apps, video, audio, social media, websites, and SMS	Digital platforms (mobile applications, videos, internet—websites or social media, SMS, etc.) to improve HIV testing uptake. This could include interventions using social media for peer support or targeted advertising, personalized web directories for HIV testing, or SMS reminders for HIV testing.

### Search strategy and inclusion criteria

The following electronic databases were searched through September 28, 2021: PubMed, PsycInfo, Cochrane CENTRAL, CINAHL Complete, Web of Science Core Collection, EMBASE, and Global Health Database. CAB Abstracts were searched through October 10, 2019; however, access to this database ceased after this date. No language restrictions were placed on the search. The following conference abstract books were searched: International AIDS Conference 2017, 2018, 2019, 2020, and 2021. The Conference on Retroviruses and Opportunistic Infections 2017, 2018, 2019, 2020, and 2021 were searched through the EMBASE database.

A search strategy was developed by ADW, IN, and DL (a research librarian), with review and adaptation from WHO HTS team members (CJ, MJ, RB, MD) and the independent methodologist (NS). It included 3 components (full strategy in S1 Appendix): (1) an HIV testing term; (2) 4 sets of terms to reflect major groups of demand creation strategies (e.g., incentives; SMS and digital individual media; community media, counseling, and other educational interventions; peer-based interventions); and (3) the sensitivity- and precision-maximizing version of the Cochrane Highly Sensitive Search Strategy for identifying RCTs [7]. The conference abstract book search terms were more limited, due to the limited search ability on conference websites, and can be found in S1 Appendix.

This review was informed by the population, intervention, comparator, outcome (PICO) question: “*Which demand creation strategies are effective for enhancing uptake of HIV testing services (HTS)*?” In order to be included in the review, studies needed to be published in a peer-reviewed journal or conference abstract, employ an RCT (either cluster- or individual-level randomization, including stepped-wedge studies), and meet PICO criteria. The population of interest was individuals receiving demand creation interventions for HTS; the interventions were those aimed to generate demand for HTS; the comparators were those with either an alternative demand creation strategy or an absence of demand creation strategies (control); the outcomes of interest included in this publication included HTS uptake (percentage of individuals who completed HIV testing among those targeted for intervention or control) and HTS yield (percentage of individuals with reactive HIV tests among those targeted for intervention or control). Yield is available only from a subset of studies and is presented when available.

### Data analysis

We used Covidence (Veritas Health Innovation, Melbourne, Australia) to manage search results and determine eligibility for the review. A series of reviewers (ADW, IN, JNE, MAB, RT, HH, NK, CW, JN, KL) were involved in screening titles and abstracts, as well as full-text articles to determine inclusion and for extraction. Two reviewers evaluated each identified abstract independently and subsequently whether records should have full-text review and abstraction; discrepancies at each step were resolved by a third reviewer. Data extraction and quality assessment were conducted by JNE, INN, JN, BM, CO, RB, KL, RT, and ADW. Two reviewers extracted data from each manuscript or abstract; at this step, the second reviewer was not blind to the extraction details of the first reviewer.

Meta-analysis using random-effects models to combine effect estimates was conducted with studies that used the same intervention and control, and outcomes were measured comparably (see details of full meta-analysis approach in S2 Appendix). Relative risks (RR), risk differences (RD), and 95% confidence intervals (CI) were calculated, along with I^2^ statistics to measure statistical heterogeneity of effect. As recommended by the Cochrane handbook, RRs were our primary estimate and RDs were considered a supporting estimate. Meta-analysis was not conducted in cases where there was clinical heterogeneity (in which the interventions compared were heterogeneous); meta-analysis was conducted and is presented at any level of statistical heterogeneity of effect. At high levels of statistical heterogeneity, subgroup analyses were conducted by region, sex, and age group. To account for clustering in cluster RCTs, the standard error of the effect estimate was inflated by multiplying it by the square root of the design effect. Meta-analysis and data summary were conducted using Stata 17 and Excel by WJ, RT, JN, and ADW.

Evidence magnitude was classified as: important: RR ≥1.5 or RR ≤0.5; less important: RR <1.5 and ≥1.25 or RR>0.5 and <0.75; and not important: RR <1.25 and >0.75. These categorizations were established with the WHO GDG to prioritize high-impact interventions for demand creation. Interpretation of effect size importance followed Effective Practice and Organisation of Care (EPOC) guidance for reporting the effects of interventions [8].

### Quality assessment

Risk of bias was assessed using the Cochrane Collaboration’s tool (version 1.0) for assessing risk of bias [9]. This tool assesses random sequence generation (selection bias), allocation concealment (selection bias), blinding of participants and personnel (performance bias), blinding of outcome assessment (detection bias), blinding of outcome assessment (detection bias), incomplete outcome data addressed (attrition bias), incomplete outcome data, and selective reporting (reporting bias). For cluster-randomized trials, an additional set of domains were assessed, based on the Cochrane handbook, including: incorrect analysis of clustered data, comparability with individually randomized trials, recruitment bias, baseline imbalances, and loss of clusters. We assessed risk of publication bias using funnel plots and Egger’s test. We conducted trim-and-fill analyses to address publication bias.

## Results

**Fig 1:**
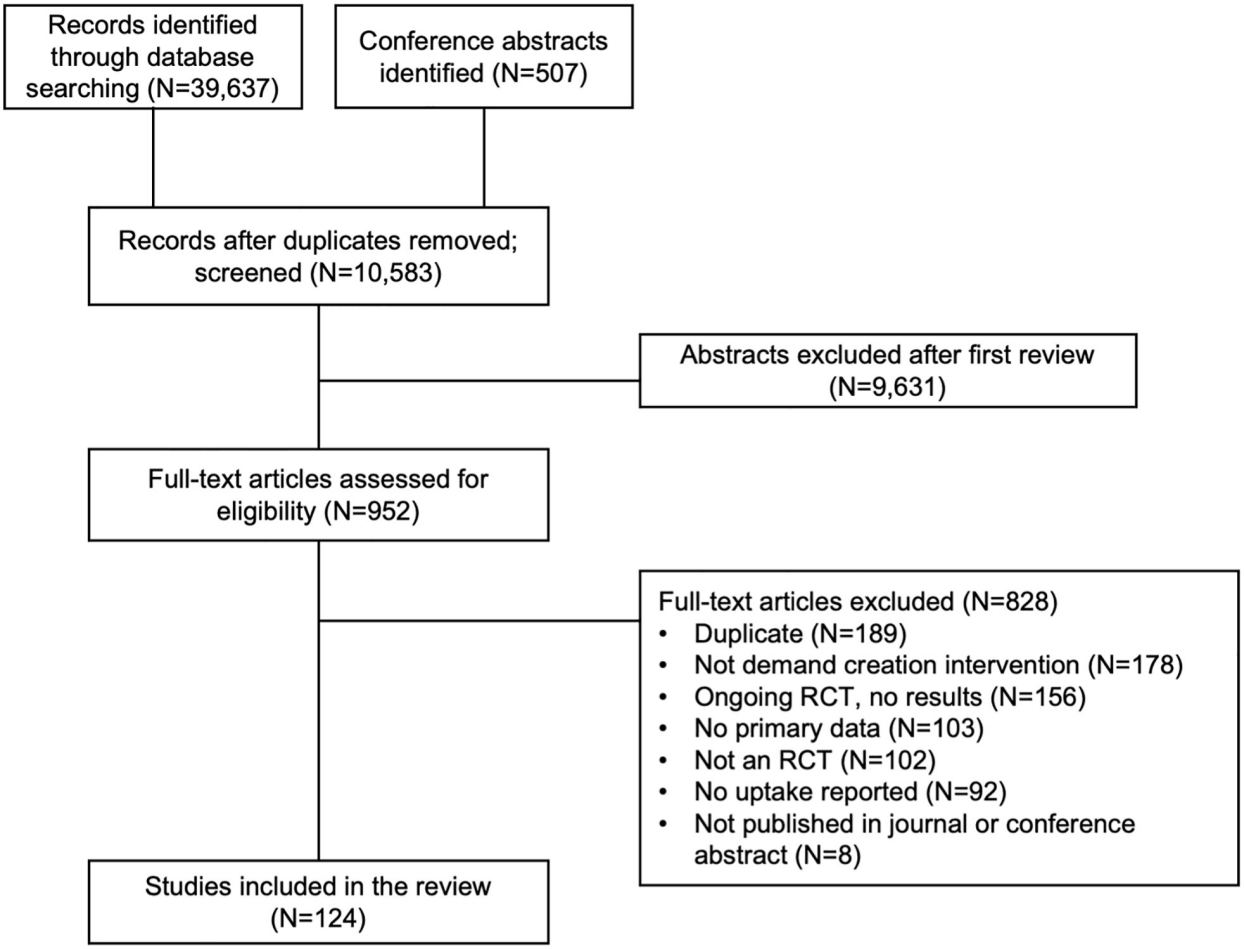
PRISMA flowchart. RCT, randomized controlled study.

The database search yielded 39,637 records and conference abstract search yielded 507 records; after duplicates were removed, 10,583 records were screened for title and abstract relevance and 9,631 were excluded as not relevant; 952 full-text articles were assessed and 828 were excluded. A total of 124 RCTs were included for data extraction; 113/124 (91%) were published peer-reviewed studies and 11/124 (9%) were conference abstracts (Fig 1); Table 1 summarizes the study characteristics. These 124 RCTs contributed data to analyses about incentives (*N* = 21), mobilization (*N* = 12), targeted and tailored interventions (*N* = 31), counseling (*N* = 39), and digital interventions (*N* = 39). RCTs could appear in multiple categories.

**Table 1:** RCTs evaluating different demand creation mechanisms.

*Author*	*Year*	*Location*	*N*	*Design*	*Population*	*Intervention mechanism*	*Comparison arm*	*Outcomes reported*	*Uptake of HTS in intervention*	*Uptake of HTS in control*	*Yield of HTS in intervention*	*Yield of HTS in control*
**Incentives to clients, *n* = 21 trials**												
Boittin [10]	2018	China	213 individuals	RCT	Sex Workers	1: Small incentive–Small pillow worth $1 2: Large incentive–Boxed set of bed sheets and pillowcases worth $15	Small incentive	Uptake of HTS	1: 106 (93%)	107 (51)		
Chamie [12]	2021	Uganda	524 individuals	RCT	Adult men and women	1: Financial incentives 2: Deposit contracts	No incentive	Uptake of HTS Yield of HTS	1: 89/172 2: 28/172	33/180		
Chamie [13]	2020	Uganda	123 individuals	RCT	Adults at increased risk (sex workers, trade center workers, people with high mobility)	1: Financial incentives–gain-framed 2: Financial incentives–commitment contract with high value 3: Financial incentives–commitment contract with low value	No incentive	Uptake of HTS	1: 22/25 2: 26/36 3: 28/38	17/24		
Chamie [11]	2018	Uganda	2,532 individuals	RCT	Adult men	1: $1 Fixed value conditional incentive + gain-framed message 2: $1 Fixed value conditional incentive + loss-framed message 3: $5 fixed value conditional incentive + gain-framed message 4: $5 Fixed value conditional incentive + loss-framed message 5: Lottery conditional incentive expected value $1 6: Lottery conditional incentive expected value $5	No control	Uptake of HTS	1: 72% 2: 75% 3: 76% 4: 78% 5: 80% 6: 76% (unclear if numerators are ITT. Denominators: 436, 408, 425, 425, 406, 427)	--	Not available by arm: 7.6% overall and ranged from 5.8% in loss-framed incentive through 9.4% in gain-framed incentive	--
Emperador [30]	2017	Uganda	602 individuals	RCT	Female partners of adult men	1: Male partner received $1 fixed value conditional incentive + gain-framed message 2: Male partner received $1 fixed value conditional incentive + loss-framed message 3: Male partner received lottery conditional incentive expected value $1	No control	Uptake of HTS	1: 117/201 (58%) 2: 124/206 (60%) 3: 123/195 (63%)	--		
Choko [14]	2021	Malawi	27 clusters; 4,544 individuals (ANC), 708 individuals (Index) ****	Cluster RCT	Adult women at first ANC visit	1: HIVST + financial incentive	HIV self-test kit alone **(SOC arm from trial not used in meta-analysis: HTS invitation for partners)	Uptake of HTS Yield of HTS	1: ANC cohort—1,000/1,632 (61.3%) 1: Index cohort—128/305 (42.0%)	ANC cohort—1,106/1,465 (75.5%) Index cohort—101/169 (59.8%)	1: ANC cohort—22/1,632 (1.3%) 1: Index cohort—32/305 (10.5%)	ANC cohort—0/1,465 (0%) Index cohort—13/169 (7.7%)
Choko [15]	2019	Malawi	71 clusters; 2,349 individuals	Cluster RCT	Adult male partners of female antenatal care attendees	1: $3 Fixed value conditional incentive + HIV self-test kit 2: $10 Fixed value conditional incentive + HIV self-test kit 3: $30 Lottery conditional incentive + HIV self-test kit (Additional trial arms not included in incentives meta-analysis: Phone call reminder + HIV self-test kit)	HIV self-test kit alone **(SOC arm from trial not used in meta-analysis: invitation letter to male partner)	Uptake of HTS Yield of HTS	1: 155/380 (40.8%) 2: 266/512 (51.9%) 3: 30/155 (19.4%) 4: 84/452 (18.6%) 5: 85/442 (19.2%)	56/408 (13.7%)	1: 11/380 (2.9%) 2: 14/512 (2.7%) 3: 4/155 (2.6%) 4: 3/452 (0.7%) 5: 11/442 (2.5%)	3/408 (0.7%)
Hawk [16]	2013	USA	149 individuals; 29 clusters	Cluster RCT	Adult women	1: $10 Fixed value unconditional incentive to attend a psychosocial intervention delivered at a party + $50 fixed value unconditional incentive to host party	Delayed intervention	Uptake of HTS Yield of HTS	45/61 (73.8%)	21/88 (23.9%)	0/61 (0%)	0/88 (0%)
Kim [17]	2016	Ethiopia	1,663 individuals	RCT	Adult men and women	2 Rounds of randomization First round of randomization 1: HIV education 2: HIV education + home-based testing 3: HIV education + variable value conditional incentive ($1.5–2.9) based on travel distance + clinic-based testing Second round of randomization 1: Variable value conditional incentive ($1.5–2.9) based on travel distance + clinic-based testing 2: Home-based testing	Standard of care	Uptake of HTS*	--	--		
Kranzer [18]	2018	Zimbabwe	2,050 households	Cluster RCT	Children and adolescents ages 8–17 years	1: $2 Fixed value conditional incentives 2: $5 or $10 lottery conditional incentive	Standard of care	Uptake of HTS	1: 316/740 (42.7%) 2: 223/661 (33.7%)	93/649 (14.3%)		
MacCarthy [19]	2020	USA	218 individuals	RCT	Latinx MSM and transgender women	1: Entered into a drawing for gift cards if tested at least once every 3 months + weekly HIV prevention text messages	Weekly HIV prevention text messages	Uptake of HTS	20/99	27/119		
Macis [20]	2021	Ecuador	46 clusters; 7,720 individuals	Cluster RCT	Adult men and women	1: Immediate incentives—$10 upon completing an HIV test 2: Delayed incentives—$10 upon collecting HIV test results 3: Behavioral nudge—Choice between making a soft commitment or a hard commitment to undergo an HIV test in the next 2 weeks	HIV Educational materials	Uptake of HTS; Yield of HTS	1: 66.34% 2. 8.72% 3. 11.01% (Denominators: 1. 805 2. 1,090 3. 3,024)	12.21% (Denominator: 2,801)	1: 0.74% 2: 0.46% 3. 0.13% (Denominators: 1. 805 2. 1,090 3. 3,024)	0.14% (Denominator: 2,801)
Maman [21]	2020	Tanzania	60 camps; 1,258 individuals	Cluster RCT	Men aged 15 and older	1: Microfinance training + peer health leadership	Standard of care	Uptake of HTS	Baseline: 45.3% Endline: 52.6% (Denominators: 621, 496)	Baseline: 46.3% Endline: 47.3 (Denominators: 627, 533)		
McCoy [22]	2013	USA	48 individuals	RCT	African American/black adult men	1: Flat incentive—$20 per seed recruitment 2: Conditional incentive- $10 per seed recruitment + bonuses	No control	Uptake of HTS*	–	–		
Montoy [23]	2018	USA	10,463 individuals	RCT	Adults in emergency department	1: $1 Fixed value conditional incentives 2: $5 Fixed value conditional incentive 3: $10 Fixed value conditional incentive	Standard of care	Uptake of HTS	1: 716/1,624 (44.1%) 2: 848/1,592 (53.3%) 3: 791/1,343 (58.9%)	2,477/5,904 (42.0%; full denominator of those randomized used)		
Njuguna [24]	2021	Kenya	452 individuals	RCT	Index case testing of children of adults living with HIV	1: $1.25 Fixed value conditional incentive + Phone call reminder 2: $2.50 Fixed value conditional incentive + Phone call reminder 3: $5 Fixed value conditional incentive + Phone call reminder 4: $10 Fixed value conditional incentive + Phone call reminder	Phone call reminder	Uptake of HTS	1: 31/89 (34.8%) 2: 44/93 (47.3%) 3: 51/92 (55.4%) 4: 54/88 (61.4%)	31/90 (34.4%)	1: 3/89 (3.4%) 2: 3/93 (3.2%) 3: 2/92 (2.2%) 4: 0/88 (0.0%)	1/90 (1.1%)
Njuguna [25]	2018	Kenya	60 individuals	RCT	Children ages 0–12 years	1: $5 Fixed value conditional incentive 2: $10 Fixed value conditional incentive 3: $15 Fixed value conditional incentive	No control	Uptake of HTS Yield of HTS	1: 15/20 (75.0%) 2: 14/20 (70.0%) 3: 15/20 (75.0%)	--	1/60 (1.7%)	
Saxena [26]	2016	USA	202 individuals	RCT	Previously incarcerated adults	1: $10 Fixed value conditional incentive	Standard of care	Uptake of HTS	1: 61/104 (59%)	46/98 (47%)		
Sibanda [27]	2017	Zimbabwe	24,679 individuals; 68 communities/clusters	Cluster RCT	Heterosexual couple testing	1: Non-financial conditional incentives of household goods valued at $1.50	Standard of care	Uptake of HTS Yield of HTS*	7,852/14,099 (55.7%)	1,062/10,580 (10.0%)		
Tanser [28]	2021	South Africa	45 clusters; 13,838 individuals	Cluster RCT	Adolescent and adult men and women	1: R50 food voucher for testing, if positive, another voucher for visiting a treatment clinic within 6 weeks 2: Male-targeted HIV-specific decision support app 3: R50 food voucher + male-targeted HIV-specific decision support app	Standard of care	Uptake of HTS	1: 680/2,481 (27.4%) 2: 566/2,120 (26.7%) 3: 430/2,534 (17.0%)	1,189/6,703 (17.7%)		
Zhou [29]	2022	China	Unknown	RCT	MSM	1: Secondary distribution with monetary incentives 2: Secondary distribution with monetary incentives + peer referral	Standard of care	Uptake of HTS*	42/101 (41.6%)	16/58 (27.6%)		
**Mobilization, *n* = 12 trials**												
Alhassan [31]	2019	Ghana	64 health facilities	Cluster RCT	Pregnant and non-pregnant women	1: Systematic community engagement in quality of health services	Standard of Care	Uptake of HTS*	Non-pregnant: Baseline: 55 (mean) Endline: 104 (mean) Pregnant: Baseline: 40 (mean) Endline: 119 (mean)	Non-pregnant: Baseline: 97 (mean) Endline: 101 (mean) Pregnant: Baseline: 101 (mean) Endline: 135 (mean)		
Berkley-Patton [32]	2019	US	4 churches; 543 individuals	Cluster RCT	Adult men and women	1: Church-based mobilization, trained peer volunteers, phone call reminders, modeling HIV testing behavior, educational videos and games, citywide mobilization, and religiously tailored and integrated messaging in sermons and in print media	Standard of care	Uptake of HTS	6 month: 47% 12 month: 59%	6 month: 28% 12 month: 42%		
Derose [34]	2016	USA	4 churches; 497 individuals	Cluster RCT	Adult men and women	1: Church based HIV education and peer leader workshops, pastor-delivered sermons on HIV with imagined contact scenarios, and HIV testing events	Standard of Care	Uptake of HTS	Latino Pentecostal church: Baseline: 7/76 (8.7%) Endline: 38.0% African American Baptist church: Baseline: 38/208 (18.1%) Endline: 32.0% (no denominator reported for endline)	Latino Pentecostal church: Baseline: 3/83 (4.1%) Endline: 7.0% African American Baptist church: Baseline: 33/130 (25.7%) Endline: 13.0% (no denominator reported for endline)		
Ezeanolue [35,36]	2015, 2017	Nigeria	40 churches; 3,040 women, 2,498 men	Cluster RCT	Adult men and women	1: Baby showers at churches as a platform for mobilizing pregnant women and their male partners to complete HIV testing	Standard of care	Uptake of HTS Yield of HTS	Men: 1,089/1,297 (84%) Women: 1,514/1,670 (91%)	Men: 453/1,201 (38%) Women 740/1,377 (54%)	Women: 41/1,670 (2.4%)	Women: 32/1,377 (2.3%)
Figueroa [37]	2010	Jamaica	50 clusters; 2,196 individuals	Cluster RCT	Adult men and women	1: Peer educator identification and encouragement to conduct group education, including condom distribution; visual media including print and video to promote condom use and take up testing; use of drama and games; and onsite HIV outreach testing	Standard of care	Uptake of HTS	367/1,535 (23.9%) Men: 173/723 (23.9%) Women: 190/812 (23.4%)	323/1,324 (24.4%) Men: 161/661 (24.4%) Women: 164/663 (24.7%)		
Indravudh [38]	2021	Malawi	30 clusters; 4,137 individuals	Cluster RCT	Adult men and women	1: Community-led HIVST intervention consisting of (i) participatory workshops for action planning with community health action groups and CHWs, (ii) trainings on HIVST promotion and support with village-level community volunteers, and (iii) HIVST campaigns linked to HIV treatment and prevention. (iv) Door-to-door distribution of HIVST kits by resident community-based distributors (CBD)	Standard of Care	Uptake of HTS	3,145/3,960 (79.4%)	1,556/3,920 (39.7%)	230/3,960 (5.8%)	
Kyegombe [39]	2014	Uganda	8 clusters; 2,240 individuals	Cluster RCT	Adult men and women	1: Community mobilization- multi-phase community activities including trainings and community activism	Delayed intervention	Uptake of HTS	Men: Baseline:39% Men: Endline: 82% (Denominators: 276, 507) Women: Baseline: 52% Women: Endline: 80% (Denominators: 254, 432)	Men: Baseline:39% Men: Endline: 54% (Denominators: 114, 404) Women: Baseline: 54% Women: Endline: 77% (Denominators: 217, 348)		
Lippman [40]	2020	South Africa	15 villages; 38,392 individuals	Cluster RCT	Adult men and women	1: Community mobilization activities to address social barriers to HIV testing and treatment	No description provided	Uptake of HTS*	–	–		
Lippman [41]	2017	South Africa	22 villages; 1,181 individuals baseline, 1,175 individuals endline	Cluster RCT	Adult men and women	1: Soccer tournaments, theater, digital story screenings, and door-to-door outreach	Standard of care	Uptake of HTS	Baseline: 60% Endline: 67%	Baseline: 63% Endline: 69%		
Sweat [42]	2011	Tanzania, Zimbabwe, Thailand	TZ: 10 clusters, 12,983 individuals Zim: 8 clusters, 22,850 individuals Thailand: 14 clusters, 21,323 individuals	Cluster RCT	Adult men and women	1: Community mobilization, mobile HTS, community-based post-test support services	Standard of care	Uptake of HTS Yield of HTS Retesting	Tanzania: 37.5% Zimbabwe: 49.9% Thailand: 69.1%	Tanzania: 8.6% Zimbabwe: 4.9% Thailand: 23.1%	Tanzania: 1.4% Zimbabwe: 6.5% Thailand 1.5%	Tanzania: 0.6% Zimbabwe: 1.1% Thailand 0.9%
Underwood [43]	2012	Zambia	34 districts; 4,816 individuals	Cluster RCT	Adult men and women	1: Mobilization—intensive 2: Mobilization—less intensive	Standard of care (no intervention)	Uptake of HTS	–	–		
**Peer-based delivery or navigation interventions, *n* = 15**												
Adam [44]	2014	Kenya	182 individuals	RCT	Adolescents and young adult students	1: Peer-delivered 32-h skills workshop	Standard of care	Uptake of HTS*	--	--		
Berkley-Patton [32]	2019	USA	4 churches; 543 individuals	Cluster RCT	Adult men and women	1: Church-based mobilization, trained peer volunteers, phone call reminders, modeling HIV testing behavior, educational videos and games, citywide mobilization, and religiously tailored and integrated messaging in sermons and in print media	Standard of care	Uptake of HTS	6 month: 47% 12 month: 59%	6 month: 28% 12 month: 42%		
Derksen [33]	2015	Malawi	122 villages	Cluster RCT	Adults	1: Emphasize personal benefits of HIV treatment and reduced transmission potential	Emphasize personal benefits of HIV treatment	Uptake of HTS*	--			
Derose [34]	2016	USA	4 churches; 497 individuals	Cluster RCT	Adult men and women	1: Church-based HIV education and peer leader workshops, pastor-delivered sermons on HIV with imagined contact scenarios, and HIV testing events	Standard of Care	Uptake of HTS	Latino Pentecostal church: Baseline: 7/76 (8.7%) Endline: 38.0% African American Baptist church: Baseline: 38/208 (18.1%) Endline: 32.0% (no denominator reported for endline)	Latino Pentecostal church: Baseline: 3/83 (4.1%) Endline: 7.0% African American Baptist church: Baseline: 33/130 (25.7%) Endline: 13.0% (no denominator reported for endline)		
Figueroa [37]	2010	Jamaica	50 clusters; 2,196 individuals	Cluster RCT	Adult men and women	1: Peer educator identification and encouragement to conduct group education, including condom distribution; visual media including print and video to promote condom use and take up testing; use of drama and games; and onsite HIV outreach testing	Standard of care	Uptake of HTS	367/1,535 (23.9%) Men: 173/723 (23.9%) Women: 190/812 (23.4%)	323/1,324 (24.4%) Men: 161/661 (24.4%) Women: 164/663 (24.7%)		
Harawa [45]	2020	USA	105 individuals	RCT	Black MSM	1: Peer mentors + incentives to clients (financial and behavioral nudges)	Incentives to clients only	Uptake of HTS; Yield of HTS	Baseline: 10/55 (18.2%) Endline: 31/55 (56.4%)	Baseline: 8/50 (16.0%) Endline: 22/50 (44.0%)	1/55 (1.8%)	2/50 (4.0%)
Outlaw [46]	2010	USA	188 individuals	RCT	MSM	1: 30-min field outreach session based on motivational interviewing with a peer outreach worker followed by offer of oral swab HIV testing 2: 30-min traditional field outreach session with a peer outreach worker followed by offer of oral swab HIV testing	No control	Uptake of HTS	47/96 (49.0%)	18/92 (20.0%)		
Rhodes [47]	2020	USA	21 social networks; 166 individuals	Cluster RCT	Immigrant, Spanish-speaking Latinx MSM and transgender women	1: Targeted peer navigation intervention- HOLA	Standard of care	Uptake of HTS	Baseline: 58.1% Endline: 90.2% (Denominators: 86, 82)	Baseline: 55.0% Endline: 60.0% (Denominators: 80, 75)		
Rhodes [48]	2017	USA	204 individuals	Individual RCT	MSM	1: Peer-delivered, 4 interactive module sessions, focused on HIV and STIs, cultural values–HOLA en Grupos	Cancer- and alcohol-related information	Uptake of HTS	Baseline: 32.45% Endline: 80.26% (Denominators: 152, 152)	Baseline: 31.58% Endline: 27.63% (Denominators: 152, 152)		
Rhodes [49]	2011	USA	142 individuals	Individual RCT	Adult heterosexually active men	1: Peer-delivered, community-based participatory research informed, intervention	Cancer-related information	Uptake of HTS	71.0%	31.6%		
Wagner [50]	2014	Senegal	64 health districts	Cluster RCT	Adults	1: Trained mentors reached out to 2 or more friends with HIV messages 2: Traditional social mobilization by community-based organization	Standard of care	Uptake of HTS*	--	--		
Wilton [53]	2009	USA	338 individuals	RCT	MSM	1: Six 2–3-h peer-led small group interventions about factors influencing HIV risk	Standard of care	Uptake of HTS Yield of HTS	69/164 (42.1%)	58/174 (33.3%)	4/164 (2.4%) (assume all new positives)	4/174 (2.3%) (assume all new positives)
Young [51]	2013	USA	4 clusters; 112 individuals	Cluster RCT	MSM	1: Facebook group with peer leaders who communicated about HIV prevention and testing and then offered a HIV home-based testing kit 2: Facebook group with peer leaders who communicated about the importance of exercising, healthy diets, and a low-stress lifestyle and then offered a HIV home-based testing kit	No control	Uptake of HTS	9/57 (15.8%)	2/55 (3.6%)		
Young [52]	2015	Peru	8 clusters; 556 individuals	Cluster RCT	MSM	1: Facebook group with peer-mentors who provided HIV prevention and behavior change information through posts and chats, emails about the importance of HIV testing and where to get tested 2: Facebook group without peer-mentors and updates about the study and HIV testing information, emails about the importance of HIV testing and where to get tested	No control	Uptake of HTS Yield of HTS*	43/278 (15.5%)	16/278 (5.8%)		
Zhou [29]	2022	China	159 individuals	RCT	MSM	1: Peer referral + secondary distribution with monetary incentives	Secondary distribution with monetary incentives only **(SOC arm from trial not used in meta-analysis)	Uptake of HTS*	Baseline: 31/50 (62.0%) Endline: 46/50 (92.0%)	Baseline: 29/50 (58.0%) Endline: 34/50 (68.0%)	Endline: 0/50 (0%)	Endline: 2/50 (4.0%)
**Personalized messages and content, *n* = 13**												
Blas [54]	2010	Peru	459 individuals	RCT	MSM	1: Online HIV-related videos tailored to gay identified MSM and non-gay identified MSM	HIV-related text	Uptake of HTS	8/142 (5.6%) gay-identified 11/97 (11.34) non-gay identified	10/130 (7.69%) gay identified 0/90 (0%) non-gay identified		
Bull [55]	2004	USA	1,776 individuals	RCT	MSM	1: Tailored HIV-related messages with accompanying photo	Non-tailored HIV-related messages	Uptake of HTS*	85%	80%		
Cordova [56]	2018	USA	50 individuals	RCT	Adolescent and young adults	1: Targeted and tailored technology-based HIV preventative mobile application	Standard of care	Uptake of HTS*	52%	45.8%		
Frye [57]	2020	USA	236 individuals	RCT	MSM and transgender people	1: Personalized HIV testing information	Standard of care	Uptake of HTS	6 months: 68/118 (57.6%)	6 months: 73/118 (61.9%)		
Haukoos [58]	2021	USA	76,561 individuals	RCT	Men and women at least 16 years of age	1: Traditional targeted HIV screening 2: Enhanced targeted HIV screening	Non-targeted HIV screening	Uptake of HTS; Yield of HTS	1: 3,137/25,639 (12.2%) 2: 4,488/25,453 (17.6%)	6,744/25,469 (26.5%)	1: 7/25,639 (0.03%) 2: 7/25,453 (0.03%)	10/25,469 (0.04%)
Horvath [59]	2020	USA	113 individuals	RCT	MSM	1: Mobile app with HIV testing recommendations, reminders, and information	Standard of care	Uptake of HTS	Month 4: 29/57 (51.9%) Month 8: 32/57 (56.1%)	Month 4: 27/56 (48.2%) Month 8: 31/56 (55.4%)		
Kalichman [61]	1993	USA	106 individuals	RCT	African-American women	1: Culturally tailored 20 min video with HIV risk-reduction messages 2: Standard video by a gender-ethnicity matched presenter	Standard video, information about HIV presented by a white male and a white female broadcaster	Uptake of HTS*	1: 156/3,028 (5.2%) 2:175/3,065 (5.7%)	172/3,187 (5.4%)		
Kalichman [60]	1995	USA	100 individuals	Cluster RCT	African-American women	1: Gender-ethnicity-matched video with HIV information, which emphasized a person-loss frame relevant to HIV testing 2: Gender-ethnicity-matched standard videos with HIV information	Ethnicity matched standard video with HIV information	Uptake of HTS*	--	--		
Luo [62]	2021	China	9,280 individuals	RCT	MSM	1: HIV risk assessment + personalized feedback based on risk score 2: HIV risk assessment only	HIV education	Uptake of HTS*	–	–		
O’Connor [63]	2014	Ireland	6,000 individuals	RCT	Adults	1: Video-based pre- and post-test counseling from a chosen counselor	Video-based pre- and posttest counseling from a single pre-assigned counselor	Uptake of HTS	2,452/2,950 (83.1%)	2,468/3,050 (80.9%)		
Sullivan [64]	2021	USA	1,229 individuals	RCT	MSM	1: Mobile app that provides prevention messages and access to core prevention services	Waitlist control	Uptake of HTS	–	–		
Tanser [28]	2021	South Africa	45 clusters; 13,838 individuals	Cluster RCT	Men > = 15 years old	1: R50 food voucher for testing, if positive, another voucher for visiting a treatment clinic within 6 weeks 2: Male-targeted HIV-specific decision support app 3: R50 food voucher + male-targeted HIV-specific decision support app	Standard of care	Uptake of HTS	1: 680/2,481 (27.4%) 2: 430/2,534 (17.0%) 3: 566/2,120 (26.7%)	1,189/6,703 (69.8%)		
Yun [65]	2021	China	192 individuals	RCT	MSM	1: WeChat delivered comprehensive HIV education package with tailored feedback on individual risk and a link to a website/online platform	Link to resources on HIV health education	Uptake of HTS	75/86 (87.2%)	68/82 (82.9%)		
**Personal invitation letters, *n* = 4**												
Byamugisha [66]	2011	Uganda	1,060 individuals	RCT	Male partners of pregnant women	1: Invitation letter addressed to male partner requesting him to accompany her to next ANC visit	Letter containing information concerning services offered at ANC	Uptake of HTS Yield of HTS	82/530 (15.5%)	68/530 (12.8%)	3/530 (0.6%)	0/530 (0%)
Vrana-Diaz [67]	2018	Kenya	1,410 individuals	RCT	Male partners of pregnant women	1: Personal invitation- improved card 2: Personal invitation + home-based test kit	Standard of care	Uptake of HTS	1: 136/467 (29.1%) 2: 334/472 (70.8%)	110/471 (23.4%)		
Mohlala [68]	2011	South Africa	1,000 individuals	RCT	Male partners of pregnant women	1: Written invitation to attend for VCT delivered by pregnant female partner	Written invitation to attend pregnancy information session	Uptake of HTS	161/500 (32.2%)	57/500 (11.4%)		
Turan [69]	2018	Kenya	127 individuals	RCT	Male partners of pregnant women	1: Letter for male partners and couple HIV testing and counseling services at the household that included health education, relationship, and communication skills	Letter for male partners	Uptake of HTS	34/64 (53.1%)	12/63 (19.0%)		
**HIV-specific information and counseling, *n* = 14**												
Booth [70]	2011	USA	632 individuals	RCT	Injection drug users	1: Two-session, HIV/HCV counseling and education model added to treatment as usual 2: A one-session, therapeutic alliance intervention conducted by outpatient counselors to facility treatment entry plus added to treatment as usual	Treatment as usual—HIV/HCV risk assessment and referral for testing and counseling	Uptake of HTS	1: 38/212 (18%) 2:12/209 (6%)	17/211 (8%)		
Dolcini [71]	2010	USA	4 clusters; 264 individuals	Cluster RCT	African-American adolescent females	1: Counseling on HIV/STI and sexual health	Counseling on diet and exercise	Uptake of HTS	(34.6%)	(33.6%)		
Festinger [72]	2016	USA	200 individuals	RCT	Adults in judicially supervised regimen of drug abuse treatment	1: 20-min-long computer-based HIV risk reduction interventions to computer-based sessions	Life skills video	Uptake of HTS	52/99 (51.5%)	35/101 (35.4%)		
Firestone [73]	2016	Liberia	60 clusters: 1,052 individuals	Cluster RCT	Adolescents and adults	1: 6-day intensive group learning intervention combined with on-site SRH services	Existing alternative education program	Uptake of HTS	Baseline: 241/639 (38%) Endline: 482/549 (88%)	Baseline: 237/518 (46%) Endline: 211/503 (42%)		
Jiraphongsa [74]	2002	Thailand	40 clusters; 400 individuals	Cluster RCT	Adults	1: Counseling in group meeting	Standard of care	Uptake of HTS	20/198 (10%)	10/200 (5%)		
Metsch [75]	2012	USA	1,281 individuals	RCT	Injection drug users	1: Brief participant-tailored risk-reduction counseling with the offer of an on-site rapid HIV test 2: Information only (description of the testing procedure) with the offer of an on-site rapid HIV test	Referral for off-site HIV testing	Uptake of HTS Yield of HTS	1: 338/433 (78.1%) 2: 347/419 (82.8%)	78/429 (18.2%)	1: 2/433 (0.46%) 2: 1/419 (0.24%)	0/429 (0%)
Diane McKee [76]	2011	USA	4 clusters; 1,043 individuals	Cluster RCT	Adolescents	1: Educational material for adolescents	Standard of care	Uptake of HTS*	Male: Baseline: 3.9% Endline: 7% Female: Baseline: 11.4% Endline: 11.8%	Male: Baseline: 1.5% Endline: 4%		
Pronyk [77]	2008	South Africa	8 clusters	Cluster RCT	Women	1: Microfinance loans and HIV and gender training	Standard of care	Uptake of HTS	28/108 (25.9%)	16/112 (14.3%)		
Simpson [78]	1999	United Kingdom	3,024 individuals	RCT	Pregnant women	1: “All blood tests” leaflet with minimal discussion protocol and offer of testing 2: “All blood tests” leaflet with comprehensive discussion protocol and offer of testing 3: “HIV-specific” leaflet with minimal discussion protocol and offer of testing 4: “HIV-specific” leaflet with comprehensive discussion protocol and offer of testing	No leaflet, no discussion protocol, no offer of testing although testing was available and advertised in a letter about the study	Uptake of HTS	1: 179/495 (36.2%) 2: 193/521 (37.0%) 3: 171/495 (34.5%) 4: 164/519 (31.6%) ***	55/994 (5.5%)		
Speizer [79]	2020	South Africa	105 schools; 2,802 individuals	Cluster RCT	Grade 8 adolescent girls	1: Tailored scripted education curriculum focused on HIV and sexual and reproductive health	Standard curriculum	Uptake of HTS; Yield of HTS	Mpumalanga endline: 332/833 (39.9%) Kwazulu-Natal endline: 271/1,040 (26.1%)	Mpumalanga endline: 246/785 (31.3%) Kwazulu-Natal endline: 213/925 (23.0%)	Mpumalanga: 35/833 (4.2%) Kwazulu-Natal: 39/1040 (3.8%)	Mpumalanga: 39/785 (5.0%) Kwazulu-Natal: 28/925 (3.0%)
Spielberg [80]	2013	India	55 clusters; 1,675 individuals	Cluster RCT	Adult and adolescent females	1: Learning Games for Girls (LGGs): introductory game, plus savings and HIV/AIDS learning games	Introductory game; no further education	Uptake of HTS	1/215 (0.4%)	2/634 (0.3%)		
Stephenson [81]	2020	USA	202 individuals	RCT	Transgender youth aged 15–24	1: Video chat HIV testing counseling	Home delivered HIVST	Uptake of HTS; Yield of HTS	59/126 (46.8%)	69/76 (90.8%)	0/126 (0%)	2/76 (2.6%)
Uhrig [82]	2012	USA	1,567 individuals	RCT	African-American women	1: Billboard ad and radio ad content	Standard of care	Uptake of HTS*				
Ybarra [83]	2017	USA	302 individuals	RCT	MSM	1: Comprehensive HIV prevention program for sexual minority males delivered via text message	Text messaging focused on general health topics (e.g., self-esteem)	Uptake of HTS	Post intervention: 28/150 (18.7%) 3 months post intervention: 38/150 (25.3%)	Post intervention: 13/152 (8.6%) 3 months post intervention: 20/152 (13.2%)		
**Couple-oriented counseling, *n* = 4**												
Chiou [84]	2015	Taiwan	84 individuals	RCT	MSM recently diagnosed with HIV	1: Two partner notification counseling sessions at HIV-positive diagnosis	Standard of care	Uptake of HTS Yield of HTS	78/226 (34.5%)	33/118 (27.9%)	31/226 (13.7%)	9/118 (77.6%)
Darbes [85]	2019	South Africa	334 couples; 668 individuals	Cluster RCT	Heterosexual couples	1: Couple-based group session followed by four couples’ counseling sessions plus monthly text reminders for HTS and HTS locations	Monthly text reminders for HTS and HTS locations	Uptake of HTS Yield of HTS	142/336 (42.3%)	40/332 (12.0%)	59/336 (17.56%)	15/332 (4.52%)
Matovu [86]	2016	Uganda	1,174 individuals	RCT	Heterosexual couples	1: Two small group interactive session (1 couple-focused + 1 male-focused session)	Standard of care	Uptake of HTS	371/697 (53.2%)	257/477 (53.9%)		
Orne-Gliemann [87]	2013	Cameroon, Dominican Republic, Georgia, India	1,943 individuals	RCT	Male partners of pregnant women	1: Couple-oriented post-test HIV counseling for pregnant women	Standard post-test HIV counseling	Uptake of HTS	Cameroon: 59/239 (24.7%) Dominican Republic: 56/242 (23.1%) Georgia: 66/246 (26.8%) India: 86/243 (35.4%)	Cameroon:35/245 (14.3%) Dominican Republic: 49/242 (20.2%) Georgia: 3/245 (1%) India: 64/241 (26.6%)		
**Message framing/content, *n* = 12**												
Apanovitch [88]	2003	USA	531 individuals	RCT	Adult women	1: 2 gain-framed videos 2: 2 loss-framed videos	None	Uptake of HTS*	--			
Brown [89]	2018	England	9,585 individuals	RCT	Individuals at high risk of HIV acquisition	1: Primer sent 1 day after dispatch plus standard reminders 2: Behavioral insights reminders (no primer) 3: Primer plus behavioral insights reminders	Standard reminders send on days 3 and 7 after dispatch of test kits	Uptake of HTS	1: 1,204/2,421(49.7%) 2: 1,222/2,357 (51.8%) 3: 1,276/2,411 (52.9%)	1,175/2,396 (49.0%)		
de Tolly [90]	2012	South Africa	2,533 individuals	RCT	Adults	1: Three SMS messages and motivational (MOTI) 2: Ten SMS messages and motivational (MOTI) 3: Three SMS messages and content (informational (INFO) 4: Ten SMS messages and content (informational (INFO)	No text	Uptake of HTS	1: 120/438 (27.4%) 2: 143/438 (32.6%) 3: 130/438 (29.7%) 4: 154/438 (35.2%)	224/801 (27.9%)		
Derksen [33]	2015	Malawi	122 villages	Cluster RCT	Adults	1: Emphasize personal benefits of HIV treatment and reduced transmission potential	Emphasize personal benefits of HIV treatment	Uptake of HTS*	--			
Exner [91]	2002	USA	341 individuals	RCT	Adult women	1: Four and 8 session sexual risk reduction intervention	Standard of care	Uptake of HTS Yield of HTS	73/233 (31.4%)	16/108 (14.8%)	0/233 (0%)	0/108 (0%)
Kasting [92]	2014	USA	2,148 individuals	RCT	Adult women	1: Message framing—one-sided messaging 2: Message framing—two-sided messaging, refuting a small objection 3: Message framing—two-sided messaging, refuting a large objection	Information-only control	Uptake of HTS	1: 80% 2: 83% 3: 82% (Denominator: 480, 481, 475)	86% (Denominator: 483)		
Kavanagh [93]	2020	Uganda	2,362 individuals	RCT	Adult men	1: Behavioral nudge—calendar with planning prompt for HIV testing	Calendar with no planning prompt	Uptake of HTS	928/1,203 (77.1%)	868/1,159 (74.9%)		
Mikolajczak [94]	2012	The Netherlands	1,704 individuals	RCT	MSM	1: Messaging avoided use of risk information and focused on advantages 2: Messaging focused on risk information and communication	No control	Uptake of HTS	98/870 (11%)	112/834 (13%)		
Patel [98]	2020	India	244 individuals	RCT	MSM	1: Approach framing (focus on positive outcome) messages 2: Avoidance framing (focus on negative outcome) messages	No control	Uptake of HTS	1: baseline: 22, endline: 26/122 (21.3%) 2: baseline: 19, endline: 31/122 (25.4%)			
Salvadori [95]	2020a	Thailand	651 individuals	RCT	Adult men and women	1: Scheduled HIV retest appointment + appointment reminder 2: Appointment reminder only	Standard of care	Uptake of HTS; Yield of HTS	1: 42/218 (19.3%) 2: 80/218 (36.7%)	24/215 (11.2%)	1: 0/218 (0%) 2: 0/218 (0%)	0/215 (0%)
Salvadori [96]	2020b	Thailand	1,895 individuals	Individual RCT	Adult men and women	1: Computer-assisted interactive counseling on a tablet computer followed by an invitation to ask questions to the counselor	Standard of Care	Uptake of HTS	63/945 (6.7%)	61/950 (6.4%)	1/945 (0.1%)	0/950 (0.0%)
Smith [97]	2021	South Africa	12 days; individuals 1,048	Cluster RCT	Adult men	1: U = U message framing	Standard of care	Uptake of HTS; Yield of HTS	112/504 (22.2%)	68/544 (12.5%)	7/504 (1.4%)	3/544 (0.6%)
**Motivation-oriented counseling, *n* = 4**												
Bentz [99]	2010	France	54 individuals	RCT	HIV risk exposure following sexual intercourse	1: Patient-centered counseling based on motivational interviewing including topics of treatment adherence and care follow up	No counseling–traditional medical management	Uptake of HTS	Testing (1.5 mo): 24/28 (85.7%) Testing (3 mo): 19/28 (67.9%)	Testing (1.5 mo): 14/26 (53.8%) Testing (3 mo): 10/26 (38.5%)		
Carey [100]	2008	USA	60 individuals	RCT	Adults seeking STI services	1: 20-min motivational interviewing	15-min information session	Uptake of HTS	1: 13/29 (44.8%)	6/31 (19.4%)	1: 0/29 (0%)	0/31 (0%)
Chang [101]	2021	Uganda	40 clusters; 2,148 individuals	Cluster RCT	Men and women ages 15–49	1: Health scouts-based motivational interviewing by mobile phone	Access to health scouts without mobile phone supported counseling	Uptake of HTS	Midline: 1,184/1,254 (94.4%) Endline: 923/944 (97.8%)	Midline: 1,225/1,279 (95.8%) Endline: 938/959 (97.8%)		
Simbayi [102]	2004	South Africa	228 individuals	RCT	Adults seeking STI services	1: 60-min motivation/skills building counseling	20-min HIV information session	Uptake of HTS	1: 35/114 (30.7%)	29/114 (25.4%)		
**Reduced duration or intensity of counseling (non-inferiority), *n* = 4**												
Cohan [103]	2009	USA	278 individuals	RCT	Pregnant women	1: Abbreviated pre-test counseling	Standard of care pre-test counseling	Uptake of HTS	133/135 (98.5%)	139/146 (95.2%)		
Diallo [104]	2010	USA	30 groups; 313 individuals	Cluster RCT	Black women	1: Interactive session	Didactic education	Uptake of HTS	32/161 (19.9%)	16/ 152 (10.5%)		
Edelman [105]	2013	USA	30 individuals	RCT	Injection drug users	1: Enhanced sexual risk management	Brief sexual risk management	Uptake of HTS	14/15 (93.0%)	12/15 (80.0%)		
Merchant [106]	2015	USA	957 individuals	RCT	Injection drug users in emergency departments	1: Motivational interviewing encased in a brief intervention	No behavioral intervention	Uptake of HTS	175/516 (33.9%)	204/514 (39.7%)		
**Video-based or audio-based, *n* = 12**												
Alemagno [107]	2009	USA	212 individuals	RCT	Adult criminal offenders under community supervision	1: Computerized self-directed intervention on a “talking laptop” using brief negotiation interviewing method	Standard of care (HIV-related text)	Uptake of HTS	27/108 (25.0%)	9/104 (8.7%)		
Aronson [108]	2021	USA	295 individuals	RCT	13–24-year-old emergency department patients	1: HIV health literacy video	Standard of care	Uptake of HTS; Yield of HTS	39/147 (26.5%)	39/148 (26.4%)	0/147 (0%)	0/148 (0%)
Blas [54]	2010	Peru	459 individuals	RCT	MSM	1: Online HIV-related videos tailored to gay identified MSM and non-gay identified MSM	HIV-related text	Uptake of HTS	8/142 (5.6%) gay-identified 11/97 (11.34) non-gay identified	10/130 (7.69%) gay identified 0/90 (0%) non-gay identified		
Calderon [109]	2007	USA	404 individuals	RCT	Adults in emergency department	1: Video-based pre-test counseling	Standard of care (counselor-led counseling)	Uptake of HTS	187/202 (92.6%)	9/202 (4.5%)		
Calderon [115]	2011	USA	200 individuals	RCT	Adolescents (15–21) attending emergency departments	1: Video-based pre-test counseling	Standard of care (counselor-led counseling)	Uptake of HTS Yield of HTS	51/100 (51.0%)	22/100 (22.0%)	0/100 (0%)	0/100 (0%)
Hirshfield [110]	2012	USA	3,092 individuals	RCT	MSM	1: Dramatic video about HIV 2: Documentary video about HIV 3: Dramatic video about HIV and a documentary video about HIV	1: A link to a website with information about HIV among MSM with links to prevention resources 2: Links to prevention resources	Uptake of HTS	1, 2, 3: 142/1,874 (7.6%) (3 video arms pooled)	1: 41/609 (6.7%) 2: 48/609 (7.9%)		
Kurth [111]	2013	USA	517 individuals	RCT	Adults	1: Provider-initiated testing and counseling using counseling provided via a computer kiosk followed by rapid testing offered by a dedicated HIV test provider staff member	Standard of care	Uptake of HTS Yield of HTS	251/258 (97.3%)	3/259 (1.2%)	1/258 (0.4%)	0/259 (0%)
Merchant [112]	2011	USA	566 individuals	RCT	Adults in emergency department	1: Audio computer-assisted interview system which assessed HIV risk behaviors and provided tailored feedback	HIV risk behavior assessment	Uptake of HTS	153/286 (53.5%)	157/285 (55.1%)		
Richens [113]	2010	UK	2,351 individuals	RCT	Individuals 16 and older attending sexual health clinic	1: Computer-assisted self-interview 2: Computer-assisted personal interview with a clinician	Standard of care (clinician completes paper form with patient)	Uptake of HTS	1: 498/801 (62.1%) 2: 512/763 (67.1%) ***	540/787 (68.6%)		
Stephenson [81]	2020	USA	202 individuals	RCT	Transgender youth aged 15–24	1: Video chat HIV testing counseling	Home delivered HIVST	Uptake of HTS; Yield of HTS	59/126 (46.8%)	69/76 (90.8%)	0/126 (0%)	2/76 (2.6%)
Wang [116]	2018	Hong Kong SAR, China	430 individuals	RCT	MSM	1: Video promoting HIV testing, self-testing and online real-time instructions and counseling, self-test kits 2: Video promoting HIV testing coupled with a list of places to get tested	No control	Uptake of HTS Yield of HTS	1: 193/215 (89.8%) 2:109/215 (50.7%)	--	1: 1/215 (0.5%)	--
Washington [114]	2017	USA	56 individuals	RCT	MSM	1: Social media posts of HIV-related videos	Social media posts of HIV-related text	Uptake of HTS	16/28 (57.1%)	8/28 (28.6%)		
**Social media-based interventions, *n* = 8**												
Horvath [59]	2020	USA	113 individuals	RCT	MSM	1: Mobile app with HIV testing recommendations, reminders, and information	Standard of care	Uptake of HTS	Month 4: 29/57 (50.9%) Month 8: 32/57 (56.1%)	Month 4: 27/56 (48.2%) Month 8: 31/56 (55.3%)		
Rhodes [117]	2016	USA	4 clusters	Cluster RCT	MSM and transgender people	1: Promoting HIV testing on social media sites by creating online profiles and making public posts	Standard of care	Uptake of HTS	63.7% (no denominator reported)	42.0% (no denominator reported)		
Sullivan [64]	2021	USA	1,229 individuals	RCT	MSM	1: Mobile app that provides prevention messages and access to core prevention services	Waitlist control	Uptake of HTS	–	–		
Tang [119]	2018	China	4 clusters; 1,381 individuals	Cluster RCT, stepped wedge	MSM, transgender people assigned male at birth	1: Direct messages on WeChat with images promoting HIV testing, social media campaign with stories of HIV testing, and a self-testing platform on WeChat (each cluster received the intervention during different 3-month periods)	Standard of care	Uptake of HTS*	Unable to extract due to nature of design	Unable to extract due to nature of design		
Washington [114]	2017	USA	56 individuals	RCT	MSM	1: Social media posts of HIV-related videos	Social media posts of HIV-related text	Uptake of HTS	16/28 (57.1%)	8/28 (28.6%)		
Young [51]	2013	USA	4 clusters; 112 individuals	Cluster RCT	MSM	1: Facebook group with peer leaders who communicated about HIV prevention and testing and then offered a HIV home-based testing kit 2: Facebook group with peer leaders who communicated about the importance of exercising, healthy diets, and a low-stress lifestyle and then offered a HIV home-based testing kit	No control	Uptake of HTS	9/57 (15.8%)	2/55 (3.6%)		
Young [52]	2015	Peru	8 clusters; 556 individuals	Cluster RCT	MSM	1: Facebook group with peer-mentors who provided HIV prevention and behavior change information through posts and chats, emails about the importance of HIV testing and where to get tested 2: Facebook group without peer-mentors and updates about the study and HIV testing information, emails about the importance of HIV testing and where to get tested	No control	Uptake of HTS Yield of HTS*	43/278 (15.5%)	16/278 (5.8%)		
Zhu [118]	2019	China	100 individuals	RCT	MSM	1: 2 HIVST kits + mobile app with HIVST promotion and HIV prevention messages	2 HIVST kits	Uptake of HTS; Yield of HTS	Baseline: 31/50 (62%) Endline: 46/50 (92%)	Baseline: 29/50 (58.0%) Endline: 34/50 (68.0%)	0/50 (0%)	2/50 (4.0%)
**Website (non-social media) based, n = 5**												
Blas [54]	2010	Peru	459 individuals	RCT	MSM	1: Online HIV-related videos tailored to gay identified MSM and non-gay identified MSM	HIV-related text	Uptake of HTS	8/142 (5.6%) gay-identified 11/97 (11.34) non-gay identified	10/130 (7.69%) gay identified 0/90 (0%) non-gay identified		
Bull [55]	2004	USA	1,776 individuals	RCT	MSM	1: Tailored HIV-related messages with accompanying photo	Non-tailored HIV-related messages	Uptake of HTS*	85% (no denominator reported)	80% (no denominator reported)		
Chiou [120]	2019	Taiwan	300 individuals	RCT	MSM	1: Mobile application that included sexual behavior information, behavior change reminders, and interactive online discussion board.	No intervention	Uptake of HTS* Yield of HTS*	--	--		
Mikolajczak [94]	2012	The Netherlands	1,704 individuals	RCT	MSM	1: Online intervention which does not use risk information or risk communication 2: Online intervention which does use risk information and risk communication	No control	Uptake of HTS	1: 98/870 (11%) 2: 112/834 (13%)			
Tang [121]	2016	China	721 individuals	RCT	MSM, transgender people assigned male at birth	1: Publicly crowdsourced video about HIV prevention 2: Video about HIV prevention created by a marketing company	No control	Uptake of HTS Yield of HTS	1: 114/352 (32.4%) 2: 111/369 (30.1%)	--	1: 36/352 (10.2%) 2: 33/369 (8.9%)	--
**SMS-based intervention, *n* = 13**												
de Tolly [90]	2012	South Africa	2,533 individuals	RCT	Adults	1: Three SMS messages and motivational content 2: Ten SMS messages and motivational content 3: Three SMS messages and informational content 4: Ten SMS messages and informational content	No text	Uptake of HTS	1: 120/438 (27.4%) 2: 143/438 (32.6%) 3: 130/438 (29.7%) 4: 154/438 (35.2%)	224/801 (27.9%)		
Govender [122]	2019	South Africa, Zimbabwe, Mozambique	1,783 individuals	RCT	Long distance truckers, sex workers, community residents	1. Text messages promoting regular HIV testing and safe sex practices	One-time verbal information about HIV testing and safe sex practices	Uptake of HTS	323/960 (33.6%)	265/823 (32.2%)		
Van Heerden [123]	2015	South Africa	90 individuals	RCT	Adult men	1: Direct message voice call 2: Direct message text message 3: Instant message	No control	Uptake of HTS	1: 3/30 (10.0%) 2: 0/30 (0%) 3: 1/30 (3.0%)			
Kelvin [124]	2019a	Kenya	2,196 individuals	RCT	Female sex workers	1: Three SMS messages that HIV self-testing is available 2: Three SMS messages that HIV testing is available 3: One SMS message that HIV self-testing is available	No control	Uptake of HTS Yield of HTS	1: 81/750 (10.8%) 2: 46/750 (6.1%) 3: 43/696 (6.2%)	--	1: 5/750 (6.2%) 2: 0/750 (0%) 3: 0/696 (0%)	
Kelvin [125]	2019b	Kenya	2,262 individuals	RCT	Truck drivers	1: Three SMS messages that HIV self-testing is available 2: Three SMS messages that HIV testing is available 3: One SMS message that HIV self-testing is available	No control	Uptake of HTS Yield of HTS	1: 26/750 (3.5%) 2: 10/750 (1.3%) 3: 10/762 (1.3%)	--	1: 5/750 (0.7%) 2: 0/750 (0%) 3: 0/762 (0%)	
Menacho Alvirio [126]	2021	Peru	400 individuals	RCT	MSM	1: Messages containing health information and topics related to HIV prevention shared with participants through WhatsApp for 12 weeks	Standard of care	Uptake of HTS	1. 82/200 (41.0%)	17/200 (8.5%)		
Mugo [127]	2016	Kenya	410 individuals	RCT	Suspected of acute HIV infection and HIV-antibody negative at baseline	1: Text messages (for those who had phones) and in-person visit (if no phone). If no response to text messages, eventual call	Given appointment cared to return in 2 weeks’ time	Uptake of HTS Yield of HTS	117/199 (58.8%)	85/211 (40.3%)	0/199 (0%)	0/211(0%)
Njuguna [128]	2016	Kenya	4 clusters; 600 individuals	Cluster RCT	College students	1: Weekly messages for 6 months about sexual health and HIV	Monthly SMS-based HIV testing surveys	Uptake of HTS	201/300 (67.0%)	155/300 (51.7%)		
Nuwamanya [129]	2020	Uganda	1,112 individuals	RCT	College students	1: Access to an interactive mobile phone app providing sexual and reproductive health information, goods, and services	Standard of care	Uptake of HTS	90.5% /556	69.6%/556		
Salvadori [95]	2020a	Thailand	651 individuals	RCT	Adult men and women	1: Scheduled HIV retest appointment + appointment reminder 2: Appointment reminder only	Standard of care	Uptake of HTS; Yield of HTS	1: 42/218 (19.3%) 2: 80/218 (36.7%)	24/215 (11.2%)	1: 0/218 (0%) 2: 0/218 (0%)	0/215 (0%)
Wettermann [130]	2019	USA	36 individuals	RCT	Adult men and women	1: HIV testing text messages	Control text message	Uptake of HTS	1: 6/17 (35.3%) 2: 0/12 (0%)	--		
Wilson [131]	2017	United Kingdom	2,063 individuals	RCT	Adolescents and adults	1: SMS link to site offering HIV self-tests	SMS with link to site with clinic information	Uptake of HTS Yield of HTS	365/1,031 (35.4%)	140/1,032 (13.6%)	0/1,031 (0%)	0/1,032 (0%)
Yun [65]	2021	China	192 individuals	RCT	MSM	1: WeChat delivered comprehensive HIV education package with tailored feedback on individual risk and a link to a website/online platform	Link to resources on HIV health education	Uptake of HTS	75/86 (87.2%)	68/82 (82.9%)		
**Gamification n = 1**												
Ybarra [132]	2021	Uganda	202 individuals	RCT	Youth aged 18–22	1: Text message based program with game-like features	Text messages about general health topics	Uptake of HTS	1: 70/101 (69.3%)	56/101 (55.4%)		

* Counts could not be extracted from the manuscript or abstract.

** Comparison arm for meta-analysis differs from SOC arm in trial.

*** Trial arms combined in meta-analysis.

**** Populations from trial included in meta-analysis as separate populations.

HTS, HIV testing services; MSM, men who have sex with men; RCT, randomized controlled trial; SMS, short message service; SOC, standard of care.

**Fig 2:**
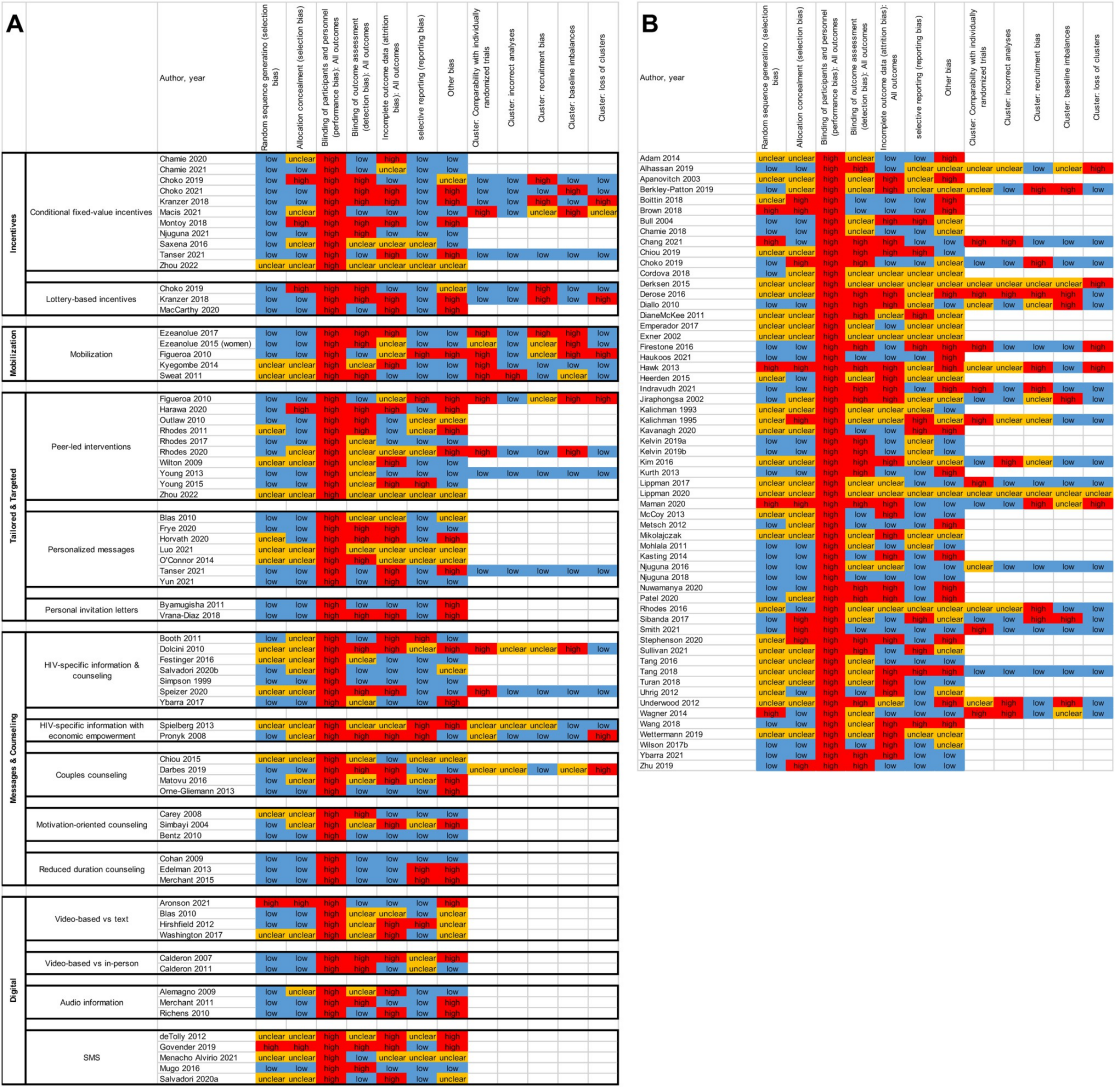
(A and B) Risk of bias summary. Panel (A): Pooled studies. Studies are grouped by the meta-analysis that they contributed to. Panel (B): Not pooled studies. Studies that were not pooled are listed in alphabetical order.

Descriptions of study categories are provided in the text box above (Box 1). Studies represented a range of regions, with 53 trials from the African region, 54 from the region of the Americas, 12 from the Western Pacific region, 9 from the European region, and 7 from the Southeast Asia region (S3 Appendix). Across studies, the risk of bias varied, with many having high or unclear risk of bias elements (Fig 2A and 2B). Among the meta-analysis pooled studies, risk of bias was medium (conditional fixed value incentives, lottery incentives, mobilization, peer-led, personalized messages, personal invitation letters, HIV-specific information and counseling, HIV-specific information with economic empowerment, couples counseling, motivation-oriented counseling, reduced duration counseling, video- and audio-based, SMS) or low (reduced duration counseling). We reviewed each study and identified 4 that had only 1 element of high risk of bias (lack of randomization). Unfortunately, none of the 4 studies tested the same intervention and it was therefore not possible to conduct a sensitivity analysis in the meta-analyses restricted to studies with low risk of bias.

### Incentives

There were 21 RCTs that tested incentives for clients or partners [10–30] (Table 1). Fourteen RCTs were conducted in Africa [11–15,17,18,21,24,25,27,28,30], 6 in the Americas [16,19,20,22,23,26], and 2 in Asia [10,29]. Seven focused on heterosexual couples [12,14,17,20,23,27,30], 4 on heterosexual men [11,15,21,22], 4 on women or caregivers of children [14,16,24,25], 2 on children and adolescents [18,28], 5 on priority populations including sex workers [10,13], previously incarcerated adults [26], and transgender women and MSM [19,29].

#### Uptake of HTS

**Fig 3:**
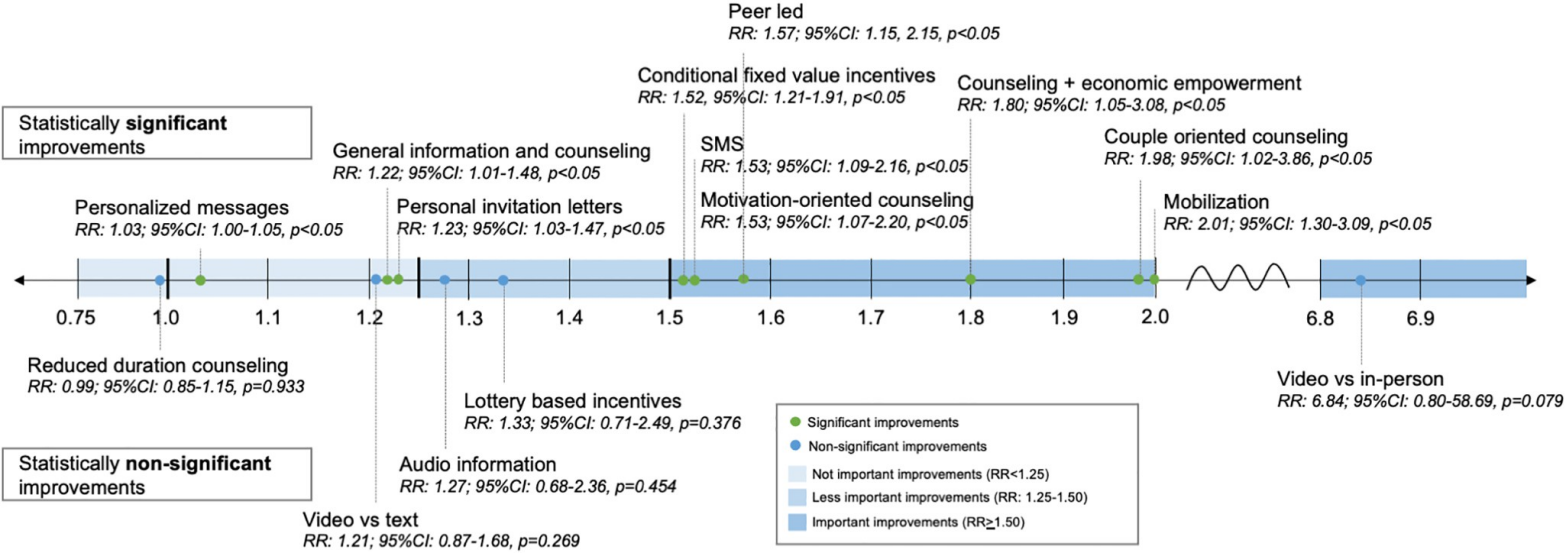
Summary of effect sizes of pooled estimates of uptake from meta-analysis. Pooled RR effect sizes ranged from near 1.0 (indicating no improvement in uptake of HTS) through nearly 7.0 (indicating a large effect size); not important improvements (RR <1.25) are shown in light blue, less important improvements (RR: 1.25–1.50) are shown in medium blue, and important improvements (RR ≥1.50) are shown in dark blue along the effect size spectrum from left to right. The statistical significance of each pooled estimate is indicated by green points (indicating statistical significance: *p* < 0.05) or blue points (indicating no statistical significance: *p* > 0.05). CI, confidence interval; HTS, HIV testing service; RR, relative risk; SMS, short message service; vs., versus.

**Fig 4:**
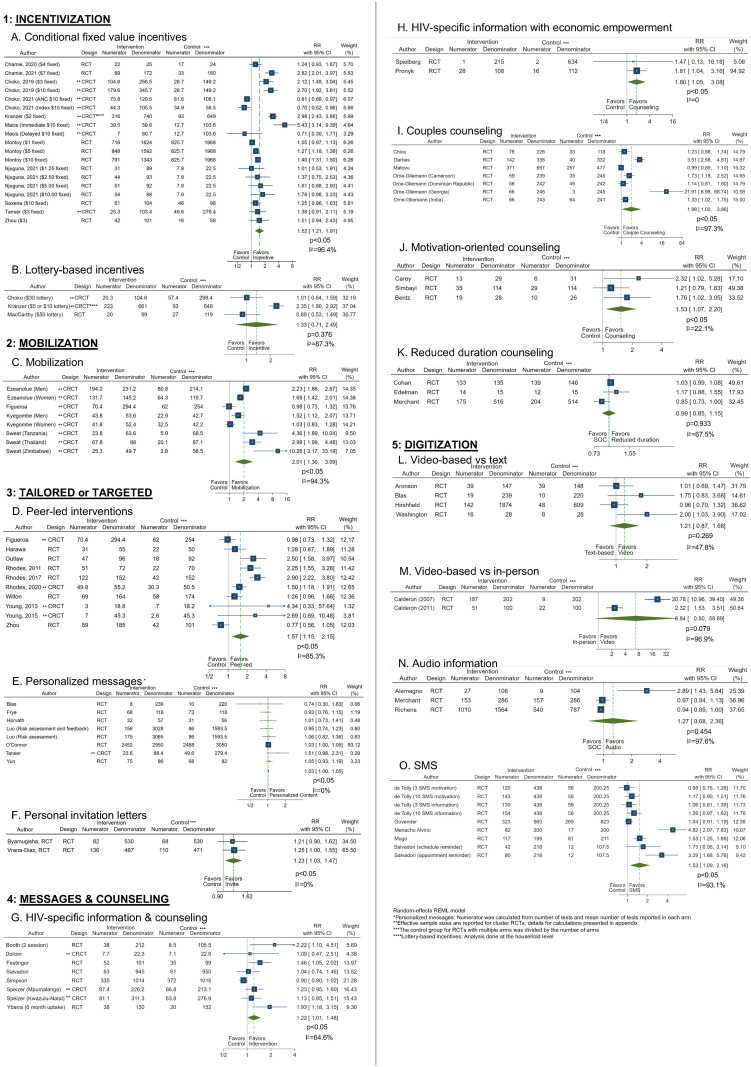
Meta-analysis plots of HTS uptake in relative and absolute differences. Panels (A–O) 1: INCENTIVES: (A) conditional fixed value incentives, (B) lottery-based incentives, 2: MOBILIZATION: (C) mobilization, 3: TAILORED or TARGETED: (D) peer-led interventions, (E) personalized messages, (F) personal invitation letters, 4: MESSAGES & COUNSELING: (G) HIV-specific information and counseling, (H) HIV-specific information with economic empowerment, (I) couples counseling, (J) motivation-oriented counseling, (K) reduced duration counseling, 5: DIGITIZATION: (L) video-based vs text, (M) video-based vs. in-person, (N) audio information, (O) SMS. CI, confidence interval; CRCT, cluster-randomized trial; RCT, randomized controlled trial; RD: risk difference; REML, restricted maximum likelihood; RR, relative risk; SMS, short message service; SOC, standard of care.

Eleven RCTs reported on uptake of HTS following use of fixed value, conditional incentives of any value compared to no incentive [12–15,18,20,23,24,26,28,29]. Fixed financial incentives significantly and importantly increased uptake of HTS compared to no incentive in the pooled analysis (pooled RR: 1.52, 95% CI [1.21, 1.91], *p* < 0.05; I^2^ = 96.4%; pooled RD: 0.15, 95% CI [0.07, 0.22], *p* < 0.05; I^2^ = 95.1%) (Figs 3 and 4 and S7 Appendix) with medium risk of bias. Three RCTs reported on uptake of HTS using lottery-based incentives of any value compared to no incentive [15,18,19]. A meta-analysis of these studies showed lottery-based incentives did not significantly but did less importantly impact HTS uptake compared to no incentive (RR: 1.33, 95% CI [0.71, 2.49], *p* = 0.376; I^2^ = 87.3%; RD: 0.06, 95% CI [−0.08, 0.20], *p* = 0.375; I^2^ = 89.5%) (Figs 3 and 4 and S7 Appendix) with medium risk of bias. Eight RCTs [10,11,16,17,21,22,25,27,30] were not pooled due to intervention and incentive heterogeneity.

In subgroup analyses by region, the effect of fixed value, conditional incentives was consistent across the African region, region of the Americas, and Western Pacific region (RR: 1.55, 1.45, 1.51, respectively). In subgroup analyses by age and sex, the effect of fixed value, conditional incentives was more pronounced among children and adolescents (RR: 1.75), women (RR: 2.41), and less pronounced among men (RR: 1.44) and trials that included men and women (RR: 1.15). While heterogeneity for lottery-based incentives was high, there were too few trials to explore subgroups (S4 Appendix).

#### Yield

**Fig 5:**
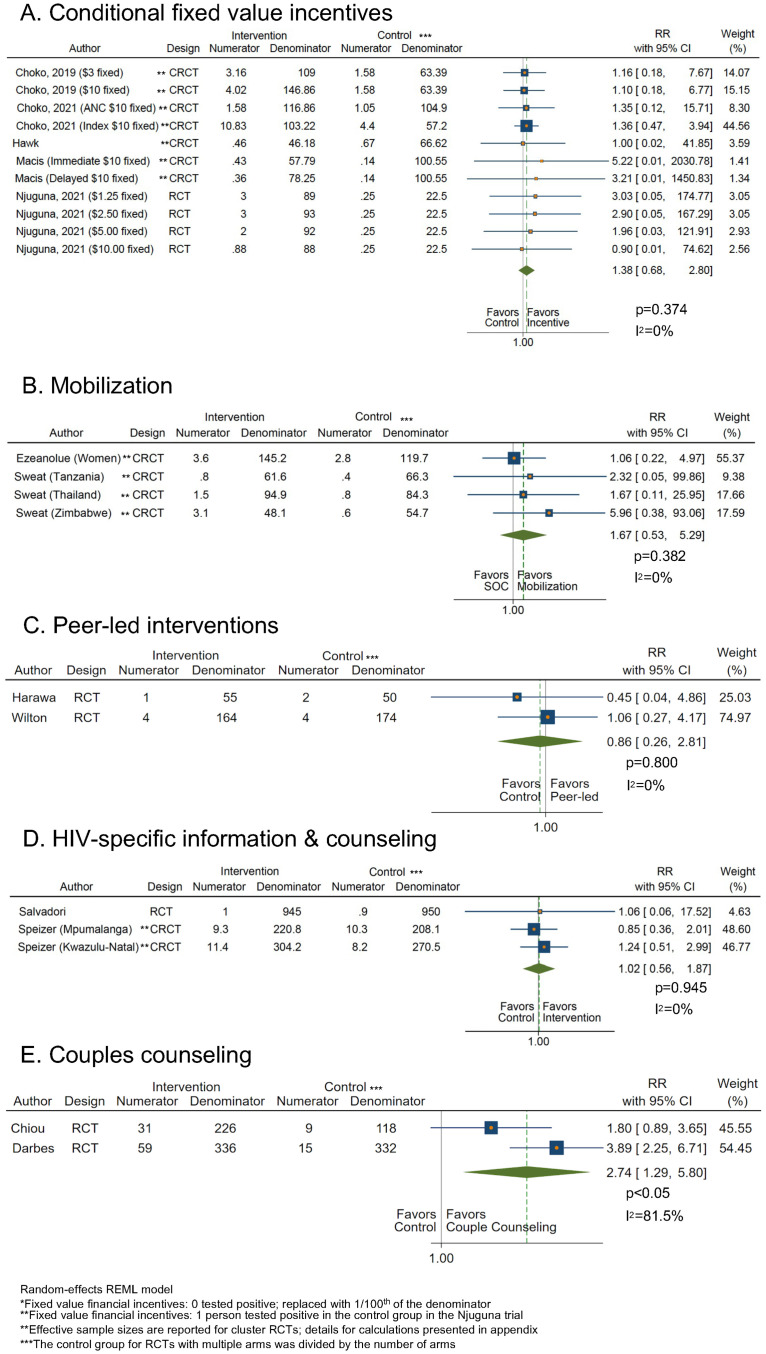
Meta-analysis plots of HTS yield in relative and absolute differences. Panels (A–E): (A) conditional fixed value incentives, (B) mobilization, (C) peer-led interventions, (D) HIV-specific information and counseling, (E) couples counseling. CI, confidence interval; CRCT, cluster-randomized trial; RCT, randomized controlled trial; RD, risk difference; REML, restricted maximum likelihood; RR, relative risk; SOC, standard of care.

Five RCTs reported on the effect of fixed value financial incentives on yield compared to no incentive [14–16,20,24]. A meta-analysis of these studies showed incentives less importantly but not significantly increased yield overall (RR: 1.38, 95% CI [0.68, 2.80], *p* = 0.374; I^2^ = 0%; RD: 0.01, 95% CI [−0.00, 0.02], *p* = 0.322; I^2^ = <0.1%) (Fig 5 and S7 Appendix). This estimate may be biased due to at least 1 zero count in the numerator.

### Mobilization

Twelve RCTs examined the role of mobilization [31–43] (Table 1). Nine RCTs were in Africa [31,33,35,36,38–43] and 3 were in the Americas [32,34,37]. Twelve focused on general population heterosexual adult men and women and while 1 focused on women.

#### Uptake

Four RCTs [35–37,39,42] reported on the effect of any mobilization on HTS uptake compared with standard HTS without mobilization. A meta-analysis of these studies showed mobilization significantly and importantly increased HTS uptake in community settings compared to no mobilization (RR: 2.01, 95% CI [1.30, 3.09], *p* < 0.05; I^2^ = 94.3%; RD: 0.29, 95% CI [0.16, 0.43], *p* < 0.05; I^2^ = 90.8%) (Figs 3 and 4 and S7 Appendix) with medium risk of bias. Eight RCTs [31–34,38,40,41,43] could not be pooled because of heterogeneity of interventions.

In subgroup analyses by region, the effect of mobilization was more pronounced among the African region (RR: 2.16) and Southeast Asian region (RR: 2.99) and less pronounced in the region of the Americas (RR: 0.98). In subgroup analyses by sex, the effect of mobilization was more pronounced among trials that included men and women (RR: 3.05), followed by men alone (RR: 1.88), followed by women alone (RR: 1.33) (S4 Appendix).

#### Yield

Two RCTs reported on the effect of mobilization on HTS yield compared to no mobilization [35,42]. A meta-analysis showed mobilization did not significantly but did importantly increase yield (RR: 1.67, 95% CI [0.53, 5.29], *p* = 0.382; I^2^ = 0%; RD: 0.01, 95% CI [−0.01, 0.03], *p* = 0.395; I^2^ = 0%) (Fig 5 and S7 Appendix).

### Targeted and tailored interventions

Thirty-two RCTs reported on the effect of targeted and tailored interventions. Fifteen RCTs assessed peer-led interventions [29,32–34,37,44–53], 13 RCTs assessed personalized content and messages [28,54–65], and 4 RCTs assessed the use of personal invitation letters [66–69].

### Peer-led interventions

Fifteen RCTs assessed peer-led interventions [29,32–34,37,44–53] (Table 1). Eleven RCTs were from the Americas [32,34,37,45–49,51–53], 3 were from Africa [33,44,50], and 1 was from China [29]. Eight RCTs focused on MSM [29,45–48,51–53], 5 focused on general population men and women [32–34,37,50], 1 focused on general population men [49], and 1 focused on adolescents [44].

#### Uptake

A meta-analysis of 10 studies [29,37,45–49,51–53] of peer-led interventions on HTS uptake showed peer-based interventions importantly and significantly increased HTS uptake compared to standard HTS without peer-based interventions (RR: 1.57, 95% CI [1.15, 2.15], *p* < 0.05; I^2^ = 85.3%; RD: 0.18, 95% CI [0.06, 0.31], *p* < 0.05; I^2^ = 90.2%) (Figs 3 and 4 and S7 Appendix) with medium risk of bias.

In subgroup analyses by region, the effect of peer-led interventions was more pronounced among the region of the Americas (RR: 1.72) and less pronounced among the Western Pacific region (RR: 0.77). In subgroup analyses by sex, the effect of peer-led interventions was more pronounced among trials that included men alone (RR: 1.68) than trials that included men and women (RR: 0.98). In trials that included men who have sex with men (and 1 trial that additionally included transgender women), the effect of peer-led interventions was RR: 1.61 (S4 Appendix).

#### Yield

Two RCTs reported on the effect of peer-led interventions on HTS yield compared to no peer-led intervention [45,53]. A meta-analysis showed peer-led interventions did not significantly or/and did not importantly impact yield (RR: 0.86, 95% CI [0.26, 2.81], *p* = 0.800; I^2^ = 0%; RD: −0.00, 95% CI [−0.03, 0.03], *p* = 0.826; I^2^ = 0%) (Fig 5 and S7 Appendix).

### Personalized content and messages

Thirteen RCTs examined the role of personalized content and messages [29,54–65]. Nine RCTs were from the Americas [54–61,64], 2 were from China [62,65], 1 was from Africa [28], and 1 was from Europe [63]. Seven RCTs focused on MSM [54,55,57,59,62,64,65], 2 focused on general population adults [58,63], 1 focused on adult men [28], 2 focused on adult women [60,61], and 1 focused on adolescents and young adults [56] (Table 1).

#### Uptake

Seven RCTs [28,54,57,59,62,63,65], most of which focused on MSM (excluding [28,63]), reported on HTS uptake using personalized content and included a standard of care arm. A meta-analysis of these studies showed tailored content messages did significantly but not importantly increase uptake (RR: 1.03, 95% CI [1.00, 1.05], *p* < 0.05; I^2^ = 0%; RD: 0.01, 95% CI [−0.01, 0.02], *p* = 0.362; I^2^ = 22.2%) (Figs 3 and 4 and S7 Appendix) with medium risk of bias. This estimate was heavily driven by 1 large study [63], which assessed personalized ethnic and gender messaging and showed a significant but not important increase in HTS uptake (RR: 1.03, 95% CI [1.00, 1.05]).

### Personal invitation letters

Four RCTs examined the role of personal invitation letters to male partners [66–69]. All studies were in Africa and focused on the male partners of pregnant women (Table 1).

#### Uptake

A meta-analysis of 2 of these studies [66,67] showed that personal invitation letters significantly but not importantly increased uptake (RR: 1.23, 95% CI [1.03, 1.47], *p* < 0.05; I^2^ = 0%; RD: 0.04, 95% CI [0.00, 0.07], *p* < 0.05; I^2^ = 0%) (Figs 3 and 4 and S7 Appendix) with medium risk of bias. Two other RCTs included invitation letters in both intervention and control [68,69].

### Counsel (information and counseling messages)

There were 38 RCTs that reported on the effect of counseling messages: 14 RCTs on general counseling messages prior to HTS [70–83], 4 RCTs on couples counseling [84–87], 12 RCTs on message and content framing prior to HTS [33,88–98], 4 RCTs on motivational counseling messages prior to HTS [99–102], and 4 RCTs on reduced duration counseling prior to HTS [103–106].

### HIV-specific counseling with and without economic empowerment

Fourteen RCTs examined the role of general HIV counseling messages and content prior to HTS [70–83] (Table 1). Eight studies were conducted in the Americas [70–72,75,76,81–83], 3 in Africa [73,77,79], 2 in Southeast Asia [74,80], and 1 in Europe [78]. One study focused on general population adults [74], 4 on women [75,77,78,80], 1 on MSM [83], 3 on PWID [70,72,75], and 5 on adolescents [71,73,76,79,81], one of which focused on transgender and non-binary adolescents [81].

#### Uptake

Among the 14 RCTs reported on HTS uptake following general HIV counseling messages [70–83], a meta-analysis of 7 RCTs [70–72,78,79,83,95] showed HIV-specific information and counseling messages prior to testing significantly but not importantly increased HTS uptake compared to standard services (RR: 1.22, 95% CI [1.01, 1.48], *p* < 0.05; I^2^ = 64.6%; RD: 0.05, 95% CI [0.00, 0.10], *p* < 0.05; I^2^ = 76.5%) (Figs 3 and 4 and S7 Appendix) with medium risk of bias. Two RCTs [77,80] reported on HTS uptake following HIV-specific counseling and economic empowerment. A meta-analysis of these studies indicates HIV-specific counseling with economic empowerment significantly and importantly increased HTS uptake (RR: 1.80, 95% CI [1.05, 3.08], *p* < 0.05; I^2^ = 0%; RD: 0.05, 95% CI [−0.06, 0.16], *p* = 0.407; I^2^ = 78.1%) (Figs 3 and 4 and S7 Appendix) with medium risk of bias. Six RCTs could not be pooled because of heterogeneity of interventions or lack of count data [73–76,81,82].

#### Yield

Two RCTs reflecting three regions reported on the effect of HIV-specific information and counseling message interventions on HTS yield [79,95]. A meta-analysis showed HIV-specific information and counseling messages did not significantly impact yield (RR: 1.02, 95% CI [0.56, 1.87], *p* = 0.945; I^2^ = 0%; RD: 0.00, 95% CI [−0.00, 0.00], *p* = 0.925; I^2^ = 0%) (Fig 5 and S7 Appendix). This estimate may be biased due to at least 1 zero count in the numerator.

### Couple-oriented counseling

Four RCTs [84–87] reported on couple-oriented counseling. Three studies were done in Africa [85–87] and 1 in East Asia [84]. One RCT focused on male partners of pregnant women [87], 1 focused on MSM [84], and 2 on heterosexual couples [85,86] (Table 1).

#### Uptake

Four RCTs [84–87] reported on HTS uptake following couple-oriented counseling. A meta-analysis of 2 of these studies showed couple-oriented counseling significantly and importantly increased HTS uptake compared with standard HTS (RR: 1.98, 95% CI [1.02, 3.86], *p* < 0.12; I^2^ = 97.3%; RD: 0.12, 95% CI [0.03, 0.21], *p* < 0.05; I^2^ = 91.0%) (Figs 3 and 4 and S7 Appendix) with medium risk of bias.

In subgroup analyses by region, the effect of couple-oriented counseling was most pronounced in 1 trial from the European region (RR: 21.91) and reasonably comparable across the other regions (RR: 1.80, 1.14, 1.33, 1.23). In subgroup analyses by sex, the effect of couple-oriented counseling was reasonably comparable between trials that included men alone (RR: 2.12) and trials that included men and women (RR: 1.84) (S4 Appendix).

#### Yield

Two RCTs reported on the effect of couple-oriented counseling on HTS yield [84,85]. A meta-analysis showed that couple-oriented counseling did significantly and importantly increase yield (RR: 2.74, 95% CI [1.29, 5.80], *p* < 0.05; I^2^ = 81.5%; RD: 0.10, 95% CI [0.03, 0.17], *p* < 0.05; I^2^ = 65.2%) (Fig 5 and S7 Appendix). It was not possible to explore heterogeneity through subgroup analyses because only 2 trials reported yield.

### Message content/framing

Twelve RCTs examined the role of message framing or specific content [33,88–91,93–98] (Table 1). Four studies were in Africa [33,90,93,97], 2 were in Europe [89,94], 3 were in the Americas [88,91,92], and 3 were in Asia [95,96,98]. Four focused on general population adults [33,90,95,96], 1 focused on high-risk individuals [89], 2 focused on general population men [93,97], 3 on general population women [88,91,92], and 2 focused on MSM [94,98]. None of the 12 RCTs could be pooled due to intervention heterogeneity. Trials compared framings such as community versus individual benefits [33], gain versus loss framing [88], motivational versus informational messages [90], risk-related versus risk agnostic framing [94], positive versus avoidance framing [98], and behavioral insights and priming [89], among others.

### Motivation-oriented messages and counseling

Four RCTs examined the role of motivational messages and counseling [99–102] (Table 1). Two trials were from Africa [101,102], 1 was from the United States [100], and 1 was from France [99]. All 4 focused on adults with risk factors such as seeking STI services or taking pre-exposure prophylaxis (PrEP).

#### Uptake

Three RCTs examined HTS uptake following motivation-oriented messages and counseling [99,100,102]. A meta-analysis of these studies showed motivation-oriented messages and counseling significantly and importantly increased HTS uptake (RR: 1.53, 95% CI [1.07, 2.20], *p* < 0.05; I^2^ = 22.1%; RD: 0.17, 95% CI [0.00, 0.34], *p* < 0.05; I^2^ = 54.1%) (Figs 3 and 4 and S7 Appendix) with medium risk of bias.

### Reduced duration or intensity of counseling (non-inferiority)

Four RCTs examined the role of reduced duration counseling to improve uptake of HIV testing [103–106] (Table 1). All 4 were from the US; 2 focused on women [103,104] and 2 focused on PWID [105,106].

#### Uptake

Three RCTs reporting on uptake were pooled [103,105,106]. A meta-analysis indicated reduced duration counseling has a similar effect on HTS uptake compared with longer duration counseling and information (RR: 0.99, 95% CI [0.85, 1.15], *p* = 0.933; I^2^ = 67.5%; RD: 0.00, 95% CI [−0.08, 0.09], *p* = 0.915; I^2^ = 72.9%) (Figs 3 and 4 and S7 Appendix), suggesting non-inferiority with low risk of bias. There were no differences between shorter intervals (Cohan and colleagues: 2- to 5-min versus 30-s counseling) [103]; multiple sessions and lengths (Edelman and colleagues: 4 sessions at 23 min each versus 2 sessions at 15 min each) [105]; and longer intervals (Merchant and colleagues: 60-min versus 34-min counseling) [106]. One RCT [104] could not be pooled due to intervention heterogeneity.

### Digital

There were 39 RCTs that reported on the effect of digital interventions: 12 were video- or audio-based [54,81,107–116], 8 used social media [51,52,59,64,75,117–119], 5 used websites [54,55,94,120,121], 13 used SMS messaging [65,90,95,122–131], and 1 used gamification [132].

### Video- or audio-based interventions

There were 12 RCTs that examined the role of video- or audio-based interventions [54,81,107–116] (Table 1). Ten were conducted in the Americas [54,57,81,107,109,111–115], 1 in Europe [113], and 1 in the Western Pacific (Hong Kong SAR) [116]. Five focused on general population adults [108,109,111–113], 4 among MSM [54,110,114,116], 1 among previously incarcerated adults on parole [107], and 2 among adolescents [81,115], the latter of which focused on transgender and non-binary adolescents.

#### Uptake

A meta-analysis of 4 studies [54,108,110,114] showed video-based interventions did not significantly and did not importantly increase HTS uptake compared to HTS with text (RR: 1.21, 95% CI [0.87, 1.68], *p* = 0.269; I^2^ = 47.8%; RD: 0.01, 95% CI [−0.02, 0.05], *p* = 0.383; I^2^ = 30.5%) (Figs 3 and 4 and S7 Appendix) with medium risk of bias.

A meta-analysis of 2 RCTs [109,115] comparing video counseling with in-person counseling showed video-based counseling importantly but not significantly increased HTS uptake compared to in-person counseling (RR: 6.84, 95% CI [0.80, 58.69], *p* = 0.079; I^2^ = 96.9%; RD: 0.59, 95% CI [0.01, 1.17], *p* < 0.05; I^2^ = 98.6%) (Figs 3 and 4 and S7 Appendix) with medium risk of bias.

A meta-analysis of 3 RCTs [107,112,113] showed audio versus in-person or text did not significantly but did less importantly increase HTS uptake (RR: 1.27, 95% CI [0.68, 2.36], *p* = 0.454; I^2^ = 97.6%; RD: 0.03, 95% CI [−0.09, 0.15], *p* = 0.620; I^2^ = 88.4%) (Figs 3 and 4 and S7 Appendix) with medium risk of bias. It was not possible to explore heterogeneity with subgroup analyses due to a small number of trials.

### Social media-based interventions

Eight RCTs [51,52,59,64,114,117–119] included a social media-based intervention (Table 1). Six were in the Americas [51,52,59,64,114,117] and 2 in Asia [118,119]. All focused on transgender women and/or MSM. Meta-analysis was not conducted, as there was only 1 RCT [117] that included a social media platform for delivering an intervention and had a control that did not include social media.

### Website (non-social media)-based interventions

Five RCTs [54,55,94,120,121] included a website-based intervention that was not on social media (Table 1). Two were conducted in the Americas [54,55], 1 in Europe [94], and 2 in Asia [120,121]. All 6 focused on transgender women and/or MSM. Meta-analysis was not conducted, as there were no RCTs that included a website platform for delivering an intervention and had a control that did not include a website.

### SMS

Thirteen RCTs [65,90,95,122–131] examined the effect of SMS (Table 1). Eight were conducted in Africa [90,122–125,127–129], 2 in the Americas [126,130], 2 in Asia [65,95], and 1 in Europe [131]. Four focused on general population adults [90,95,127,130], 1 focused on general population men [123], 3 focused on individuals considered to be at high risk including female sex workers and truck drivers [122,124,126], 2 on MSM [65,126], 2 on young people in university [128,129], and 1 on both adolescents and adults [131].

#### Uptake

A meta-analysis of 5 RCTs [90,95,122,126,127] showed SMS significantly and importantly increased HTS uptake (RR: 1.53, 95% CI [1.09, 2.16], *p* < 0.05; I^2^ = 93.1%; RD: 0.11, 95% CI [0.03, 0.19], *p* < 0.05; I^2^ = 90.2%) (Figs 3 and 4 and S7 Appendix) with medium risk of bias. Eight RCTs were not poolable [65,123–125,128–131]*)*.

In subgroup analyses by region, the effect of SMS was most pronounced in 1 trial from the region of the Americas (RR: 4.82), followed by the Southeast Asian region (RR: 2.40), and finally in the African region (RR: 1.16) (S4 Appendix).

#### Yield

Three RCTs [95,127,131] reported on yield following SMS compared to no SMS. A meta-analysis was not conducted as none of the studies identified any individuals living with HIV; the numerators were all zero counts.

### Publication bias

Using Egger’s tests and funnel plots (S5 Appendix), we observed no evidence of publication bias for incentives (both uptake and yield), peer-led interventions (uptake), personalized letters (uptake), motivation-oriented counseling (uptake), reduced duration counseling (uptake), mobilization (yield), HIV counseling without economic empowerment (yield), and SMS (yield). However, there appeared to be evidence of publication bias for lottery incentives (uptake), mobilization (uptake), HIV counseling without economic empowerment (uptake), couples counseling (uptake), audio versus text formats (uptake), and SMS (uptake). Due to a small number of studies, publication bias could not be assessed for invitation letters (uptake), counseling with economic empowerment (uptake), video versus in-person formats (uptake), peer-led interventions (yield), and couples counseling (yield).

We present trim-and-fill adjusted RR estimates for yield and uptake in S6 Appendix. Adjusted RR estimates generally did not differ substantially from unadjusted estimates, with the following exceptions; the association of mobilization with uptake was attenuated (unadjusted RR: 2.01 versus adjusted RR: 1.79) and with yield was attenuated (unadjusted RR: 1.67 versus adjusted RR: 1.18); the association of peer-led interventions with uptake was attenuated (unadjusted RR: 1.57 versus adjusted RR: 1.42) and with yield was enhanced (unadjusted RR: 0.86 versus RR: 1.06); the association of general counseling with yield was reversed and enhanced (unadjusted RR: 1.02 versus adjusted RR: 0.85); the association of couples counseling and yield was enhanced (unadjusted RR: 2.74 versus adjusted RR: 3.89); the association of motivational interviewing and uptake was attenuated (unadjusted RR: 1.53 versus adjusted RR: 1.21); the association of video versus text counseling and uptake was attenuated (unadjusted RR: 1.21 versus adjusted RR: 1.09) and between video versus in-person and uptake was attenuated (unadjusted RR: 6.84 versus adjusted RR: 2.32); the association of SMS and uptake was attenuated (unadjusted RR: 1.53 versus adjusted RR: 1.23).

## Discussion

In this large systematic review and meta-analysis assessing strategies to increase demand for HTS, we found that mobilization, couple-oriented counseling, peer-led interventions, motivation-oriented counseling, SMS, and conditional fixed value incentives all significantly and importantly (≥50% increase) increased HTS uptake. Lottery-based incentives and audio-based interventions less importantly (25% to 49% increase) but not significantly increased HTS uptake. Personal invitation letters and personalized message content significantly but not importantly (<25% increase) increased HTS uptake. Reduced duration counseling had comparable performance to standard duration counseling and video-based interventions were comparable or better than in-person counseling. Pooled estimates reflected trials with an average medium risk of bias, limiting the strength of conclusions drawn. Finally, message framing, social media interventions, website-based interventions, and gamification had no pooled estimates of effect due to heterogeneity in interventions or lack of a control group without some version of the intervention.

Our findings are similar to other systematic reviews of demand creation interventions for VMMC and family planning, supporting the use of incentives, mobilization, and interpersonal communication with or without peer involvement. One systematic review of demand generation strategies for VMMC highlighted that financial incentives produced the largest relative improvements and were most acceptable, followed by multicomponent mobilization efforts including education, counseling, and influencers [133]. A separate systematic review delving into different types of incentives for VMMC identified that fixed-value financial incentives were effective, while lottery-based incentives were not [134]. A third recent review echoed the prior findings, noting that conditional cash incentives and food or transport vouchers were especially effective and acceptable and that lottery incentives and gifts and subsidies were not effective for VMMC [135]. A systematic review of demand generation strategies for family planning and contraception observed that demand generation activities—including community- and facility-based interventions, financial incentives, and mass media—were associated with increased uptake of family planning; they noted that financial incentives in particular were effective [136]. A separate systematic review of family planning strategies observed that demand generation interventions like mass media and interpersonal communication, peer-led interpersonal communication, incentives, and savings groups increased utilization of family planning services [137]. Similar to family planning, demand creation may be especially important for HTS when new products are introduced in a particular setting, such as the introduction of HIV self-tests [138].

We found that mobilization efforts increase HTS uptake. Mobilization may be particularly well suited to settings and time periods where overall coverage of HTS is lower, motivating large groups within a community to take up testing by shifting the acceptability of HTS. However, as different settings approach the first 95 of the UNAIDS 95-95-95 goals in a particular population, the remaining untested individuals will be systematically different from those tested previously. Interventions that have been previously successful to motivate testing early in a population—such as mobilization—may have diminishing returns over time. This may require either tailoring to subpopulations—including key populations and their partners—or replacement with other targeted demand creation interventions. Pairing demand creation models with additional theoretical insights—such as those from the diffusion of innovations theory—may allow for more thoughtful and effective tailoring of demand creation strategies as HIV testing approaches saturation in a given population. For example, peer-led interventions had an important and significant pooled effect and most of the individual trials focused on transgender populations and men who have sex with men. This is an area for prioritization that serves priority populations and may be a focused alternative to broad and unfocused mobilization.

Provision of information and counseling messages continue to be important; we found evidence that couples counseling and motivation-oriented counseling approaches are effective. Couples counseling is effective and widespread in many contexts [139]; motivation-oriented counseling is less broadly scaled and may merit further expansion; economic empowerment interventions may have broad reaching effects beyond HTS [140]. However, individualized messaging—both personalized messages and personal invitation letters—had minimal impact on uptake of HTS and may be considered for de-prioritization.

While incentives were found to be effective in our review and others, and acceptable to end users and health care workers [141,142], acceptability to policymakers and implementers may be limited based on affordability and concerns related to longer-term sustainability. Concerns exist about erosion of intrinsic motivation for testing, although the limited data that exist do not support this concern [143].

This review highlighted a wide range of digital interventions, with substantial heterogeneity in both intervention design, impact, and gaps in the evidence related to effectiveness. Generally, older technology—such as videos and SMS—had more available evidence supporting effectiveness, while newer technology—including websites and social media and gamification—had less available evidence. This review included individual studies about social media and website-based interventions; however, we were unable to pool results due to the small number of studies with an adequate control for comparison. A 2018 review of digital interventions explored multiple attributes of digital interventions—including modality, directionality, tailoring, phrasing, and schedule—to identify what makes a digital intervention work [144].

SMS was effective for enhancing uptake of HTS in this review; a related systematic review of strategies to promote frequent HIV retesting demonstrated that SMS was effective [145]. Like other digital platforms, SMS in and of itself is less characteristic of an intervention and more a platform to deliver information or an intervention. mHealth and eHealth literature has demonstrated that reminder SMS messages are relatively ineffective, but SMS that deliver motivational or informational content or theory-informed content is more effective, particularly SMS platforms that offer interactive instead of one-way communication [146,147]. In this review, included studies ranged in their content and format; de Tolly and colleagues tested different numbers of messages and either motivational or informational content, Nuwamanya and colleagues tested an interactive mobile phone app and Yun and colleagues linked to interactive content on a website through text messages, Salvadori and colleagues sent appointment reminders, and several studies tested informational messages without interaction, which the literature suggests are least likely to produce a desired health action.

This review offered 2 opportunities for enhancing the efficiency of HTS in the face of limited resources while maintaining uptake. We did not detect any differences in the effectiveness of shorter versus longer counseling sessions; additionally, video-based information was as effective (and possibly more effective based on effect size) than in-person counseling sessions. Creation of videos may be expensive, but the one-time costs of creating a video are borne upfront with limited costs for continued use; additionally, crowdsourcing development of messaging could increase affordability. Videos offer a range of benefits for resource-limited settings: videos can be displayed in waiting rooms or on individual tablets, accommodating a range of existing infrastructure contexts; videos can be translated to different languages; offer consistent and accurate information; can be updated more rapidly than large numbers of health care workers can be trained; and can be time-saving for health care workers to shift from providing standardized pre-test information to providing individualized post-test counseling. In the era of Coronavirus Disease 2019 (COVID-19), video-based services have increased in prevalence and reach. Reducing length and intensity of counseling or shifting to a video format may make HTS implementation more feasible in many settings, particularly in contexts with flat or decreasing budgets for HTS.

This review is the largest and most comprehensive systematic review of demand creation for HTS. Its interim findings were used to inform the WHO 2019 HTS guidelines to direct global policy and this updated review was utilized in the 2023 HTS guidelines to provide better guidance on best practices. This large body of literature from the field of HIV may also be applicable to generating demand for testing services for other disease areas, serving as indirect evidence in guideline development [148].

This review was limited in several ways. It is possible that with a strict RCT string present in the search strategy, we missed some relevant RCTs; however, this Cochrane string has been well validated, and the number of citations identified without this string would have been unfeasible to manage with consistent accuracy. By restricting to RCTs, we excluded potentially informative studies that utilize non-randomized study designs; often, these designs have higher external validity than RCTs, which can provide a more accurate understanding of real world effectiveness outside of ideal trial conditions. It was not feasible to include non-randomized designs in a review of this breadth, but future research on specific types of demand creation interventions should consider including non-randomized designs. Key populations may be underrepresented in this review. We restricted our meta-analysis and pooling to studies that were randomized trials and that had the intervention tested against a standard of care that did not include the intervention. This type of evidence may be systematically less readily available for key populations; for example, studies that utilized a digital component were more likely to be unpoolable because there was a digital intervention both in the intervention and control arms and digital interventions were also more likely to focus on transgender populations and men who have sex with men. Future reviews focusing on key populations and demand creation should consider including study designs beyond randomized trials and to include gray literature. Finally, a substantial number of meta-analyses demonstrated evidence of publication bias, including 3 interventions we concluded had both an important and significant impact on HTS, including mobilization, couples counseling, and SMS. It is possible that if publication bias were not present, these interventions would not be concluded as impactful and recommended in this review.

Our meta-analyses pooled approximately half of studies included in this systematic review; categories of demand creation interventions represented by a single study were included and reported in this review, but not compared directly to the pooled estimates, nor categorized by effect size or statistical significance. Additionally, many studies tested multicomponent interventions, which are not reflected well in this type of meta-analysis; we aimed to identify the largest component of each intervention and group the trials accordingly. Review platforms that intentionally enumerate all components of an intervention or strategy and pool accordingly may reflect this nuance more precisely [149]. The quantitative pooling approach of a meta-analysis using relative risks, a relative measure, and pooled risk differences that are agnostic to HTS coverage in control groups makes it less possible to assess how the impact of interventions varies across settings with differing coverage of HTS among populations and regions. Similarly, statistical heterogeneity of effect and heterogeneity of intervention were both strongly present in this review across many intervention areas. We have provided estimates of statistical heterogeneity of effect, which appeared predominantly due to heterogeneity of setting and with context-specific details provided in data tables. This heterogeneity may make it more challenging for implementers to select context-relevant evidence; future reviews may consider making use of context heterogeneity present across trials to be informative using transportability principles [150].

Estimates of yield of testing can be considered either among the full denominator randomized or those tested. Using the full denominator randomized preserves the benefits of randomization but may combine the mixed effect of uptake and underlying prevalence; using the denominator of those tested loses the benefits of randomization but isolates the effect of prevalence among the tested population. We presented results using the full randomized denominators to preserve the benefits of randomization. Finally, the risk of bias across the included trials was medium, which weakens the strength of inference drawn. All trials were marked down in alignment with Cochrane guidance for lack of blinding of participants to their intervention arm; however, this is necessary for any demand creation intervention to have an effect and may be an artificially large mark down for this body of literature.

This large systematic review and meta-analysis provides evidence for several demand creation strategies to increase uptake of HTS. Conditional fixed value incentives, mobilization, couple-oriented counseling, motivation-oriented counseling, and SMS all significantly and importantly (≥50% increase) increased HTS uptake. Reduced duration counseling and video-based counseling can increase efficiency without reducing uptake. These specific demand creation interventions should be prioritized for programmatic consideration alongside important risks and benefits, as well as context-specific factors.

## Supporting information

S1 PRISMA checklist Prisma checklist. (DOCX)

S1 Appendix Full search strategy in PubMed format. (DOCX)

S2 Appendix Meta-analysis methods. (DOCX)

S3 Appendix Geographic distribution of included trials. Key and bar chart identify the total number of trials included from each country on the map. The rworldmap [cran.r-project.org] package in R was used to obtain the publicly available map (South A (2011). “rworldmap: A New R package for Mapping Global Data.” The R Journal, 3(1), 35–43. ISSN 2073-4859); the base layer map file can be found: https://code.google.com/archive/p/rworld/source/default/source.
(DOCX)

S4 Appendix Subgroup analyses for meta-analyses with high statistical heterogeneity. (DOCX)

S5 Appendix Funnel plots and Egger’s tests to assess publication bias. (DOCX)

S6 Appendix Trim-and-fill adjusted estimates of uptake and yield. (DOCX)

S7 Appendix Risk difference plots. (DOCX)

## Abbreviations

ART: antiretroviral therapy
CI: confidence interval
COVID-19: Coronavirus Disease 2019
EPOC: Effective Practice and Organisation of Care
GDG: Guideline Development Group
HTS: HIV testing services
MSM: men who have sex with men
PICO: population, intervention, comparator, outcome
PLWH: people living with HIV
PrEP: pre-exposure prophylaxis
PWID: people who inject drugs
RCT: randomized controlled trial
RD: risk difference
RR: relative risk
SMS: short message service
UN: United Nations
VMMC: voluntary medical male circumcision
WHO: World Health Organization
