# Supplementary material for: Demand creation for HIV testing services: A systematic review and meta-analysis
Source: PLoS Med. 2023 Mar 21;20(3):e1004169. doi: 10.1371/journal.pmed.1004169 (PMC10030044; doi:10.1371/journal.pmed.1004169)
Supplement: S1 Appendix — (DOCX) [file pmed.1004169.s002.docx]

**Appendix 1:** Full search strategy in PubMed format

*Note that while the strategy is split into 3 components, it was executed together as one search (e.g. Component 1 AND (Component 2a OR Component 2b OR Component 2c OR Component 2d), conducted at the same time.*

Component 1: HIV testing

("HIV Infections/diagnosis"[Mesh] OR "AIDS Serodiagnosis"[Mesh])

OR

(("HIV Infections"[Mesh] OR “HIV”[mesh] OR HIV[tiab] OR HIV1[tiab] OR HIV2[tiab] OR “human immunodeficiency virus” [tiab] OR “human immunedeficiency virus” [tiab] OR “human immuno deficiency virus” [tiab] OR “human immune deficiency virus” [tiab] OR ((human immun*[tiab]) AND (deficiency virus[tiab])) OR "Acquired Immunodeficiency Syndrome"[Mesh] OR ((acquired immun*[tiab]) AND (deficiency syndrome[tiab])) OR “acquired immunodeficiency syndrome”[tiab] OR “acquired immunedeficiency syndrome”[tiab] OR “acquired immuno-deficiency syndrome”[tiab] OR “acquired immune-deficiency syndrome”[tiab])

AND

("Diagnostic Tests, Routine"[Mesh] OR "Mass Screening"[Mesh] OR diagnos*[tw] OR serodiagnos*[tw] OR test[tiab] OR tests[tiab] OR testing[tiab] OR screening[tiab]))

AND

Component 2a: Incentives

("Reward"[Mesh] OR “Remuneration”[Mesh] OR “Health services needs and demand/economics”[Mesh] OR “Health promotion/economics”[Mesh] OR “Economics, medical”[Mesh] OR “Education/economics”[Mesh] OR “Financial Support”[Mesh] OR “health services accessibility/economics”[Mesh] OR "Financing, Personal"[Mesh] OR Reimburs*[tiab] OR demand [tiab] OR demands[tiab] OR incentiv* [tiab] OR payment* [tiab] OR voucher* [tiab] OR payment [tiab] OR lottery[tiab] OR lotteries[tiab] OR “cash”[tiab] OR compensat*[tiab] OR reward*[tiab] OR prize*[tiab] OR remunerat*[tiab] OR “cash transfer”[tiab] OR “cash transfers”[tiab] OR monetary[tiab] OR money[tiab] OR “financial compensation”[tiab] OR “economic compensation”[tiab] OR “performance based”[tiab])

OR

Component 2b: SMS and digital individual media

("Cell Phone"[Mesh] OR "Computers, Handheld"[Mesh] OR “Internet”[mesh] OR “Wireless Technology”[mesh] OR “Telephone”[mesh] OR "Social Media"[Mesh] OR "Electronic Mail"[Mesh] OR "Text Messaging"[Mesh]

OR

“social media”[tiab] OR “social networking”[tiab] OR crowdsourc*[tiab] OR texting[tiab] OR “text message”[tiab] OR “text messages”[tiab] OR “text messaging”[tiab] OR “short messaging service”[tiab] OR “short message service”[tiab] OR “sms message”[tiab] OR “sms messages”[tiab] OR “sms messaging”[tiab] OR “instant message”[tiab] OR “instant messages”[tiab] OR “instant messaging”[tiab] OR “phone”[tiab] OR “phones”[tiab] OR “mobile technology”[tiab] OR “mobile device”[tiab] OR “mobile devices”[tiab] OR “cellular technology”[tiab] OR smartphone*[tiab] OR telephone*[tiab] OR internet[tiab] OR wireless[tiab] OR whatsapp[tiab] OR twitter[tiab] OR video[tiab] OR videos[tiab] OR android[tiab] OR “operating system”[tiab] OR “mobile health”[tiab] OR mhealth[tiab] OR “m health”[tiab] OR ehealth[tiab] OR “e health”[tiab] OR computer[tiab] OR computers[tiab] OR website*[tiab] OR “web site”[tiab] OR “web sites”[tiab] OR internet[tiab] OR online[tiab] OR “online chat”[tiab] OR “chat room”[tiab] OR “chat rooms”[tiab] OR email[tiab] OR “e mail”[tiab] OR “electronic mail”[tiab] OR facebook[tiab])

OR

Component 2c: Community media, counseling and other educational

("Directive Counseling"[Mesh] OR "Counseling"[Mesh] OR “Drama”[mesh] OR “Mass Media”[mesh] OR “Health Fairs”[mesh] OR “Pamphlets”[mesh] OR “Communications Media”[mesh:noexp] OR “Community Networks”[mesh] OR “Social Media”[mesh]

OR

mobiliz*[tiab] OR mobilis*[tiab] OR drama[tiab] OR theater*[tiab] OR theatre*[tiab] OR campaign*[tiab] OR “health fair”[tiab] OR “health fairs”[tiab] OR sport[tiab] OR sports[tiab] OR game[tiab] OR games[tiab] OR sporting[tiab] OR congregation*[tiab] OR mosque*[tiab] OR church*[tiab] OR video[tiab] OR videos[tiab] OR videogame*[tiab] OR “community organization”[tiab] OR “community organizations”[tiab] OR “community based”[tiab] OR community event*[tiab] OR “community network”[tiab] OR “community networks”[tiab] OR community program*[tiab] OR educational program*[tiab] OR educational event*[tiab] OR educational material*[tiab] OR pamphlet*[tiab] OR ad[tiab] OR ads[tiab] OR advert[tiab] OR adverts[tiab] OR advertis*[tiab] OR banner*[tiab])

OR

Component 2d: Peer-based

("Patient Navigation"[Mesh] OR “Social Support”[mesh]

OR

mentor* [tiab] OR norm[tiab] OR norms [tiab] OR norming[tiab] OR navigation[tiab] OR “self help”[tiab] OR peer[tiab] OR peers[tiab] OR friend[tiab] OR friends[tiab] OR “social network”[tiab] OR “social networks”[tiab] OR “support group”[tiab] OR “support groups”[tiab])

AND

Component 3: Randomized trials – sensitivity and precision

((randomized controlled trial[pt] OR controlled clinical trial[pt] OR randomized[tiab] OR placebo[tiab] OR clinical trials as topic[mesh:noexp] OR randomly[tiab] OR trial[ti]) NOT (animals[mh] NOT humans [mh]))

**Conferences**

Only the term “randomi*” was used to search conference abstracts because all conferences being searched were HIV-related and search functions are limited.
