# Supplementary material for: Demand creation for HIV testing services: A systematic review and meta-analysis
Source: PLoS Med. 2023 Mar 21;20(3):e1004169. doi: 10.1371/journal.pmed.1004169 (PMC10030044; doi:10.1371/journal.pmed.1004169)
Supplement: S2 Appendix — (DOCX) [file pmed.1004169.s003.docx]

**APPENDIX 2:** Meta-analysis methods

Data were analyzed according to coding categories and outcomes. Where there were multiple studies reporting the same outcome, meta-analyses were conducted using random-effects models to combine effect estimates. When multiple study designs and/or multiple effect estimates were found, effect estimates were combined into a single pooled estimate; separate pooled estimates were calculated where either clinical or design incomparability were present. For randomized trials, the “intention-to-treat” effect estimate was used. In cases where the numerator of interest was zero (0), this count was replaced by one-thousandth of the denominator, to allow for meta-analysis pooling. In cases where multiple intervention arms shared the same control arm and were included in the same meta-analysis, the control arm’s numerator and denominator were divided by the number of intervention arms in the given meta-analysis to avoid overly precise estimates of standard error in the pooled analysis.

When outcomes were dichotomous, we calculated risk ratios (RRs) (i.e., where the number of outcomes is divided by the total number at risk and compared between exposed and unexposed as a ratio) and risk differences (RDs) for absolute differences. We presented findings both in summary tables and graphically using forest plots.

We included cluster-randomized trials in meta-analyses if sufficient information was provided to inflate the reported standard error to account for clustering. If the study reported an effect estimate that did not account for clustering, we attempted to collect the following information:

· Number of clusters (or groups) randomized to each intervention group

· Mean number of individuals in each cluster

· Outcome data ignoring the cluster design (e.g., effect estimate, number or proportion of individuals with events, or means and standard deviations)

· Estimate of the intracluster/intraclass correlation coefficient (ICC)

We inflated the standard error for effect estimates from randomized trials that were analyzed as if clustering were not present. First, we calculated the design effect (D) using the formula: *D = 1 + (M-1) ICC,* where M is the mean number of individuals per cluster. In the event that the ICC was not reported in the study manuscript, we estimated the ICC by averaging the ICCs from the other cluster randomized trials included in the review. We inflated the standard error of the effect estimate by multiplying it by the square root of the design effect. The inflated standard error was used in the meta-analysis. In the meta-analysis, the effective sample size – calculated by dividing the sample size by the calculated design effect – was reported for cluster randomized trials.

Random effects restricted maximum likelihood models were used to estimate summary treatment effects. Effect estimates from each study were weighted by the inverse of the individual study variance, which was calculated as the sum of the within-study (sampling) variance and an estimate of the between-study variance. Meta-analyses were conducted with STATA 17 using the random-effects restricted maximum likelihood models.
