# Supplementary material for: Demand creation for HIV testing services: A systematic review and meta-analysis
Source: PLoS Med. 2023 Mar 21;20(3):e1004169. doi: 10.1371/journal.pmed.1004169 (PMC10030044; doi:10.1371/journal.pmed.1004169)

**APPENDIX 3:** Geographic distribution of included trials. Key and bar chart identify the total number of trials included from each country on the map. The rworldmap [[cran.r-project.org](http://cran.r-project.org/)] package in R was used to obtain the publicly available map (South A (2011). “rworldmap: A New R package for Mapping Global Data.” The R Journal, 3(1), 35-43. ISSN 2073-4859); the base layer map file can be found: <https://code.google.com/archive/p/rworld/source/default/source>.

Location of number of trials


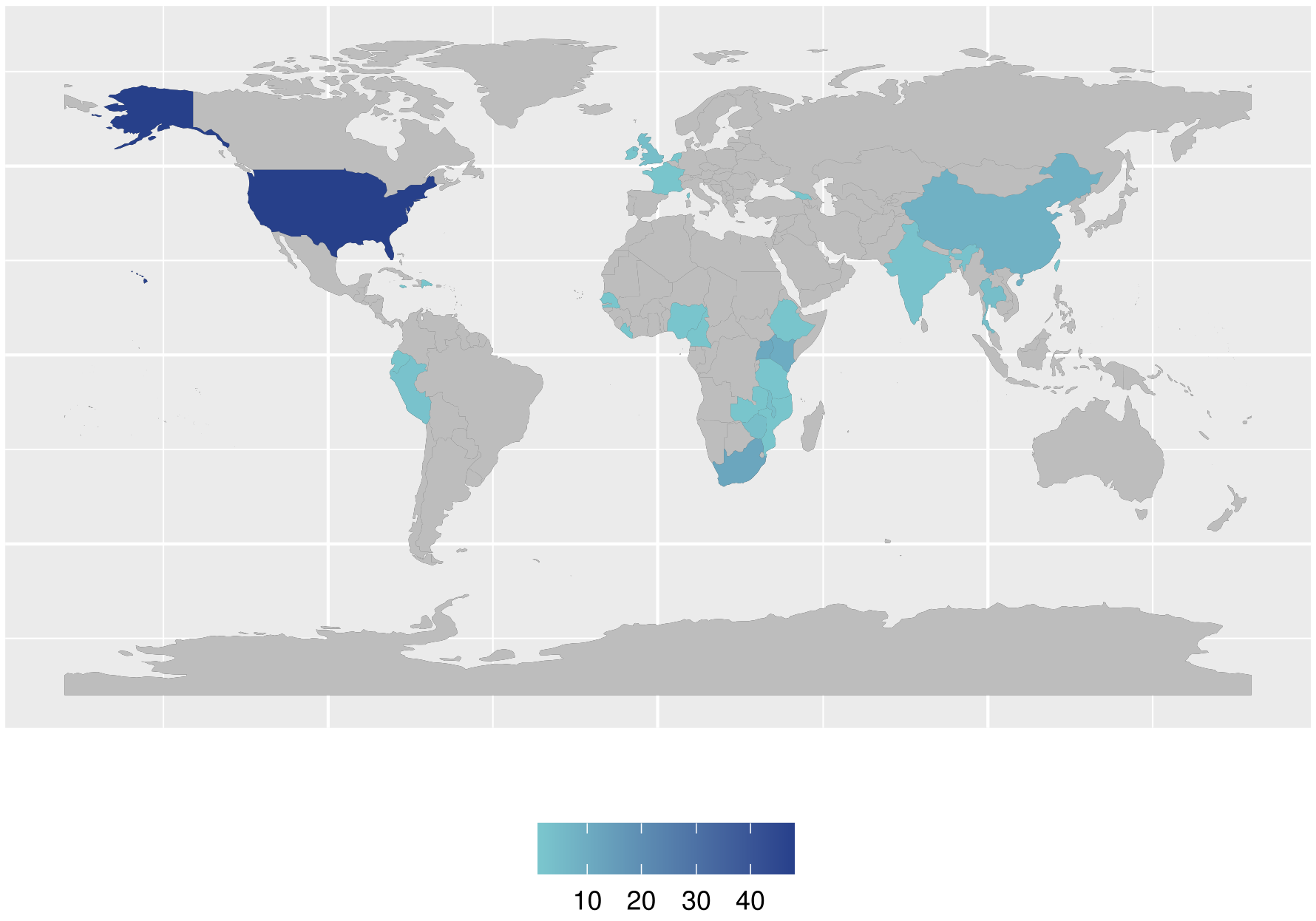

Supplement: S3 Appendix — Key and bar chart identify the total number of trials included from each country on the map. The rworldmap [cran.r-project.org] package in R was used to obtain the publicly available map (South A (2011). “rworldmap: A New R package for Mapping Global Data.” The R Journal, 3(1), 35–43. ISSN 2073-4859); the base layer map file can be found: https://code.google.com/archive/p/rworld/source/default/source. (DOCX) [file pmed.1004169.s004.docx]
