# Supplementary material for: Demand creation for HIV testing services: A systematic review and meta-analysis
Source: PLoS Med. 2023 Mar 21;20(3):e1004169. doi: 10.1371/journal.pmed.1004169 (PMC10030044; doi:10.1371/journal.pmed.1004169)
Supplement: S4 Appendix — (DOCX) [file pmed.1004169.s005.docx]

**Appendix 4.** Sub-group analyses for meta-analyses with high statistical heterogeneity

**Incentives**

**A. Region**

**B. Population: Men/Women/Children**

**Mobilization**

**A. Region**

**B. Population**

**Peer-led intervention**

**A. Region**

**B. Population: Men vs. Women**

**C. Population: All Men vs. Men who have sex with men* vs. Men and Women**

*Rhodes 2020 also includes transgender women

**Couples counseling**

**A. Region**

**B. Population: Men vs Women**

**SMS**

**A. Region**
