# Supplementary material for: Demand creation for HIV testing services: A systematic review and meta-analysis
Source: PLoS Med. 2023 Mar 21;20(3):e1004169. doi: 10.1371/journal.pmed.1004169 (PMC10030044; doi:10.1371/journal.pmed.1004169)
Supplement: S5 Appendix — (DOCX) [file pmed.1004169.s006.docx]

**Appendix 5.** Funnel plots and Egger’s tests to assess publication bias

**Incentives**

Egger’s test: p=0.7134

**Lottery incentives**

Egger’s test: p<0.0001

**Mobilization**

Egger’s test: p=0.0025

**Peer led**

Egger’s test: p=0.2582

**Personalized letters**

Egger’s test: p=0.9777

**Invitation letters**

Egger’s test: Could not be calculated

**HIV counseling without economic empowerment**

Egger’s test: p=0.0001

**HIV counseling with economic empowerment**

Egger’s test: Could not be calculated

**Couples counseling**

Egger’s test: p=0.0001

**Motivation counseling**

Egger’s test: p= 0.1384

**Reduced duration counseling**

Egger’s test: p= 0.7790

**Video vs text**

Egger’s test: p= 0.0236

**Video vs in-person**

Egger’s test: Could not be calculated

**Audio vs text**

Egger’s test: p= 0.0042

**SMS**

Egger’s test: p= 0.0015

**Funnel plots for yield**

**Incentives**

Egger’s test: p=0.6446

**Mobilization**

Egger's test p= 0.4489

**Peer led**

Egger’s test: Could not be calculated

**HIV counseling without economic empowerment**

Egger’s test: p= 0.9702

**Couples counseling**

Egger’s test: Could not be calculated

**SMS**

Egger’s test: p>0.999
