# Supplementary material for: Demand creation for HIV testing services: A systematic review and meta-analysis
Source: PLoS Med. 2023 Mar 21;20(3):e1004169. doi: 10.1371/journal.pmed.1004169 (PMC10030044; doi:10.1371/journal.pmed.1004169)
Supplement: S6 Appendix — (DOCX) [file pmed.1004169.s007.docx]

**APPENDIX 6:** Trim-and-fill adjusted estimates of uptake and yield

| **Uptake** |  |
| --- | --- |
| Fixed incentives | 1.52 (1.21-1.91) |
| Lottery incentives | 1.33 (0.71-2.49) |
| Mobilization | 1.79 (1.07-2.98) |
| Peer-led | 1.42 (1.03-1.96) |
| Personalized messages | 1.03 (1.00-1.05) |
| Invitation | 1.25 (1.07-1.45) |
| General counseling | 1.18 (0.97-1.43) |
| HIV-specific counseling and economic empowerment | 1.82 (1.07-3.07) |
| Couples counseling | 1.99 (1.02-3.86) |
| Motivation counseling | 1.21 (0.82-1.79) |
| Reduced duration counseling | 0.99 (0.86-1.16) |
| Video vs text | 1.09 (0.74-1.60) |
| Video vs in-person | 2.32 (0.20-27.43) |
| Audio vs text | 1.27 (0.68-2.36) |
| SMS | 1.23 (1.13-1.34) |
| **Yield** |  |
| Fixed incentives | 1.30 (0.65-2.80) |
| Mobilization | 1.18 (0.42-3.28) |
| Peer-led | 1.06 (0.37-3.06) |
| General counseling | 0.85 (0.52-1.38) |
| Couples counseling | 3.89 (1.67-9.04) |
