# Supplementary material for: Demand creation for HIV testing services: A systematic review and meta-analysis
Source: PLoS Med. 2023 Mar 21;20(3):e1004169. doi: 10.1371/journal.pmed.1004169 (PMC10030044; doi:10.1371/journal.pmed.1004169)
Supplement: S7 Appendix — (DOCX) [file pmed.1004169.s008.docx]

**APPENDIX 7**: Risk difference plots.

**8a:** Meta-analysis plots of HTS uptake in absolute differences. Panels A-O: 1: INCENTIVES: A) Conditional fixed value incentives, B) Lottery-based incentives, 2: MOBILIZATION: C) Mobilization, 3: TAILORED or TARGETED: D) Peer-led interventions, E) Personalized messages, F) Personal invitation letter, 4: MESSAGES & COUNSELING: G) HIV-specific information & counseling, H) HIV-specific information with economic empowerment, I) Couples counseling, J) Motivation-oriented counseling, K) Reduced duration counseling, 5: DIGITIZATION: L) Video-based vs text, M) Video-based vs in-person, N) Audio information, O) Short message service (SMS). CRCT: cluster randomized trial; RCT: randomized controlled trial; RD: risk difference; CI: confidence interval; SOC: standard of care; REML: restricted maximum likelihood

**8b:** Meta-analysis plots of HTS yield in absolute differences. Panels A-F: A) Conditional fixed value incentives, B) Mobilization, C) Peer-led interventions, D) HIV-specific information & counseling, E) Couples counseling. CRCT: cluster randomized trial; RCT: randomized controlled trial; RD: risk difference; CI: confidence interval; SOC: standard of care; REML: restricted maximum likelihood
